# A Regulatory Potential of the *Xist* Gene Promoter in Vole *M. rossiaemeridionalis*

**DOI:** 10.1371/journal.pone.0033994

**Published:** 2012-05-11

**Authors:** Konstantin E. Orishchenko, Sophia V. Pavlova, Eugeny A. Elisaphenko, Vladimir V. Sherstyuk, Alexander V. Prinz, Alexander I. Shevchenko, Elena V. Dementyeva, Suren M. Zakian

**Affiliations:** 1 Institute of Cytology and Genetics, Russian Academy of Sciences, Siberian Branch, Novosibirsk, Russia; 2 Institute of Chemical Biology and Fundamental Medicine, Russian Academy of Sciences, Siberian Branch, Novosibirsk, Russia; 3 State Research Institute of Circulation Pathology, Novosibirsk, Russia; Pennsylvania State University College of Medicine, United States of America

## Abstract

X chromosome inactivation takes place in the early development of female mammals and depends on the *Xist* gene expression. The mechanisms of *Xist* expression regulation have not been well understood so far. In this work, we compared *Xist* promoter region of vole *Microtus rossiaemeridionalis* and other mammalian species. We observed three conserved regions which were characterized by computational analysis, DNaseI *in vitro* footprinting, and reporter construct assay. Regulatory factors potentially involved in *Xist* activation and repression in voles were determined. The role of CpG methylation in vole *Xist* expression regulation was established. A CTCF binding site was found in the 5′ flanking region of the *Xist* promoter on the active X chromosome in both males and females. We suggest that CTCF acts as an insulator which defines an inactive *Xist* domain on the active X chromosome in voles.

## Introduction

Dosage compensation in female mammals is achieved by inactivation of one of the two X chromosomes. X-inactivation occurs in early embryogenesis and comprises several stages such as counting of the X-chromosome number per diploid autosome set, choice of the X-chromosome to be inactivated, initiation of inactivation, spreading of the inactive state, and its maintenance in cell lineage. X-inactivation is controlled by a locus referred to as the X chromosome inactivation center, XIC. It contains several non-coding RNA genes, most importantly *Xist* and *Tsix*. *Xist* is expressed from the inactive X chromosome. Further, *Xist* RNA spreads along the inactivating X chromosome, leading to its heterochromatinization and gene silencing [1], [2], [3].

Before the onset of X-inactivation, *Xist* is expressed at a low level from both X chromosomes. Then XICs of two X chromosomes transiently associate and the mutually exclusive choice of the future active and inactive X chromosomes occurs [4], [5], [6]. As a result, the *Xist* allele on one X chromosome is up-regulated, triggering the X-inactivation process, whereas *Xist* expression on the other X chromosome is repressed. The mechanisms underlying such expression regulation have not been well understood. In rodents, *Xist* expression on the active X chromosomes is repressed through antisense transcription of *Tsix* across the *Xist* promoter [7], [8], [9]. However, there is no *TSIX* in the human XIC [10], [11], [12]. Therefore, regulation of *Xist* expression seems to be more complicated and *Xist* promoter may possess some elements which can both activate and repress its transcription. To date *Xist* promoter region was studied only in two mammalian species, human and mouse. A number of binding sites for widespread transcription factors (TBP, YY1, SP1, CTCF) and uncharacterized regulatory proteins were found within *Xist* minimal promoter [13], [14], [15], [16], [17].

Common voles of the genus *Microtus* are characterized by interspecific differences in X chromosome morphology (size, heterochromatin blocks, and positions of centromeres). Moreover, X-inactivation in these hybrid females is skewed. The *M. arvalis* X chromosome remains active in 80% of the cells in the *M. arvalis*×*M. rossiaemeridionalis*, *M. arvalis*×*M. transcaspicus*, and *M. arvalis*×*M. kirgisorum* hybrids [18]. The mechanism of skewed X-inactivation is still unclear. However, interspecific changes in DNA sequences influencing different transcription levels of the *Xist* alleles may be involved [19], [20]. These properties make common voles an interesting model for studying the X-inactivation process.

In the four closely related common vole species the sequences of XIC elements and their expression pattern were previously analyzed [21], [22]. It has been shown that not all functional elements of the mouse XIC are well conserved even within one order Rodentia. This suggests a taxon-specific regulation of X-inactivation and the genes involved in this process.

This work was focused on studying the *Xist* promoter region of *M. rossiaemeridionalis* and searching for common and species-specific regulatory proteins which could influence *Xist* expression. We identified factors being potential activators and repressors of *Xist* expression at different stages of X-inactivation. CpG methylation of the promoter region demonstrated playing an important role in *Xist* regulation. In addition, we were not able to detect a CTCF binding site in the vole *Xist* minimal promoter which is well known in human and mouse. CTCF binding was found in the 5′ flanking region of the *Xist* promoter on the active X chromosome in both males and females, allowing us to suggest that CTCF is an insulator which defines an inactive *Xist* domain on the active X chromosome in voles.

## Results

### Comparative Analysis of *Xist* 5′ Regulatory Region in Mammals

**Figure 1:**
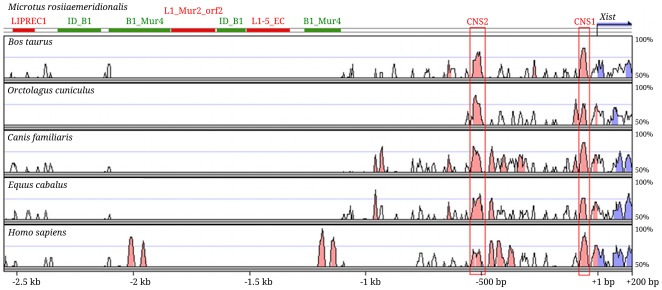
mVista plot of *Xist* 5′ end and upstream sequences based on LAGAN alignment with reference sequence *Microtus rossiaemeridionalis*. The horizontal and vertical axes represent the position in the sequences (in base-pairs) and the percent identity of the two sequences (50–100%), respectively. Parameters: Calc Window – 20 bp, Min Cons Width – 20 bp. Regions in which the identity is higher than or equal to 70% are colored in pink (non-coding regions), or blue (coding region).

The nucleotide sequences of 5′ regulatory regions and parts of the first exon of *Xist* were compared in different mammalian lineages. A comparison of *Xist* 5′ regions between *M. rossiaemeridionalis* and other mammals revealed a homology of the sequences in the −1 kb to +1 bp region. The homology was then interrupted with species-specific SINE and LINE mobile elements (Fig. 1). A more detailed analysis of the −1 kb to +1 bp region using the software searching for local conserved DNA sequences (mVista) identified two most conserved regions, CNS1 [−73/−44 bp] and CNS2 [−540/−498 bp] (conserved non-coding sequence; Fig. 1).

CNS1 was found in the *Xist* minimal promoter [−101/+1 bp]. Its homology varied from 73% for vole/horse to 97% for cow/dog with an average level of 84%. Such a high homology in the non-coding gene suggests an important role of this region in *Xist* expression regulation. Two short sequences conserved for all the species were also identified in the minimal promoter. One of them (T-T-A-A-A-G/A) is located 25 bp upstream of the transcription start site and is likely to interact with TBP-like protein [16]. The second sequence (G-C-C-A-T-G/A-T-T-T) spans the *Xist* transcription start site and seems to bind to the initiator protein YY1 [17].

CNS2 was located at positions from −540 to −498 bp relative to the vole *Xist* transcription start site (Fig. 1, Fig. S1). The homology of this region varied from 74% for human/rabbit to 93% for human/dog with an average level of 79%. Several other conserved regions were found near CNS2 almost in all the species analyzed except for cow and rabbit.

**Figure 2:**
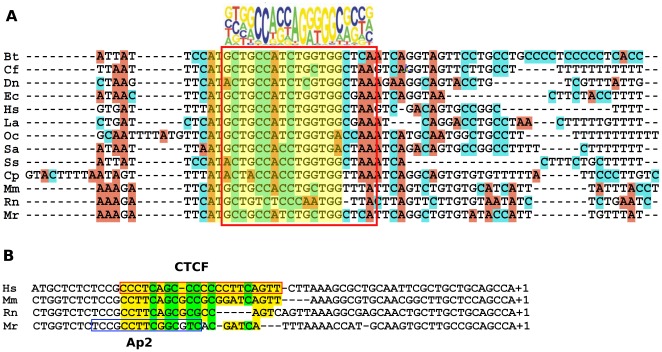
(A) Multiple alignment of CNS3 sequences of *Xist* 5′ regulatory regions in different mammalian lineages. (Bt) *Bos taurus*; (Cf) *Canis familiaris*; (Dn) *Dasypus novemcinctus* (armadillo); (Ec) *Equus caballus* (horse); (Hs) *Homo sapiens*, (La) *Loxodonta africana* (elephant); (Oc) O*ryctolagus cuniculus* (rabbit); (Sa) *Sorex araneus* (shrew); (Ss) *Sus scrofa* (pig); (Cp) *Cavia porcellus* (guinea pig); (Mm) *Mus musculus*; (Rn) *Rattus norvegicus*; (Mr) *M. rossiaemeridionalis* (vole). Putative CTCF binding sites are shown with yellow frames. Consensus of CTCF binding site is present [60]. (B) Alignment of *Xist* minimal promoter. Human CTCF is shown with red frames [14]. Vole AP2 binding site is shown with blue frame.

In addition, using multiple alignment of *Xist* promoter region of 13 mammals from different taxa we observed a short but well conserved region, CNS3 (Fig. 2A). Its localization depended on species-specific mobile elements ([−964/−944 bp] for vole *Xist*, [−1026/−1006 bp] for mouse *Xist*, [−3007/−2987 bp] for human *XIST*, [−4371/−4391] for cow *Xist*). Note that CNS3 contains a potential CTCF binding site detected by MatInspector with high expected value.

### Search for Potential Regulatory Elements in the *Xist* Promoter Region

To identify regions interacting with regulatory proteins CNS1, CNS2, and the adjacent nucleotide sequences were analyzed by DNase I *in vitro* footprinting. Two *Xist* promoter regions [−267/+54 bp] and [−551/−372 bp] were labeled with [γ^_32^P]ATP and used in footprinting reactions. The nuclear extracts from female vole fibroblast cell culture were added to binding reactions.

**Figure 3:**
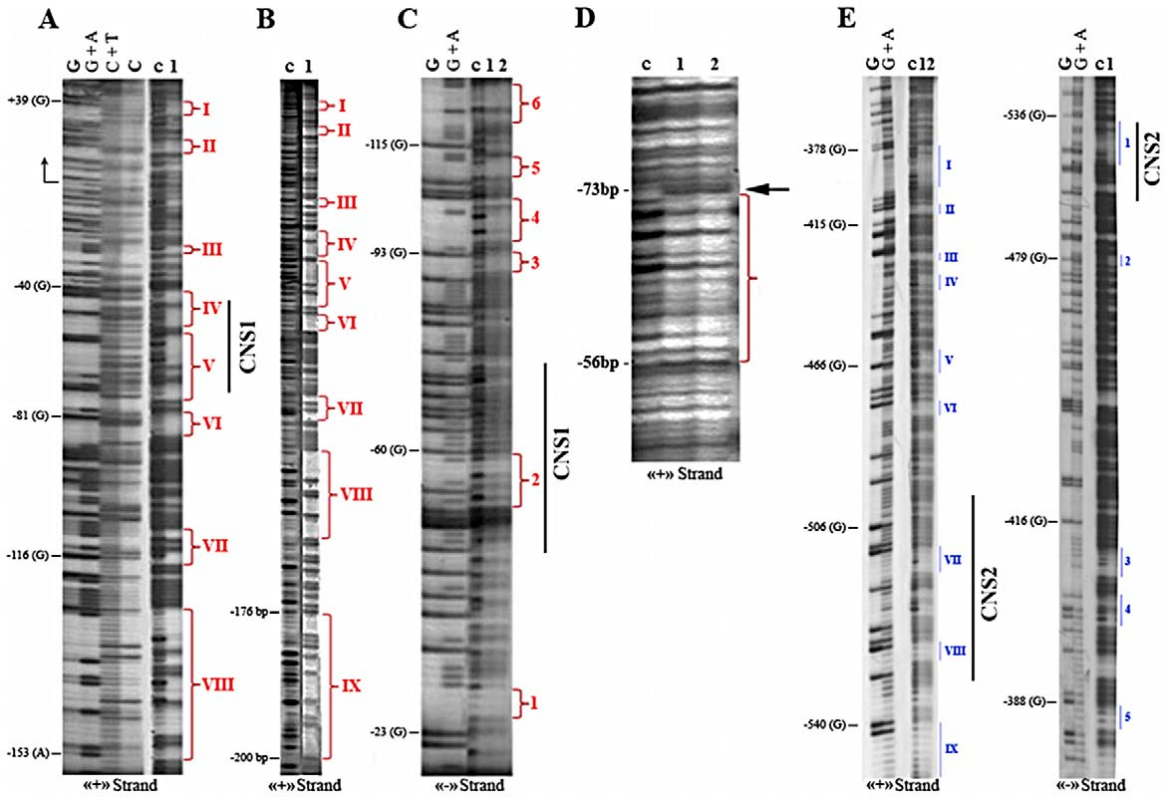
DNAse I *in vitro* footprinting analysis of the 5′-region of the vole *Xist* gene. (A) and (B) footprinting of the «+» strand of the minimal promoter. (C) «−» strand. Lane C is a control digestion without nuclear extract. Nuclear extracts were used in binding reactions: 5 µg (lane 1) and 10 µg (lane 2). (D) Footprinting with the recombinant transcription factor SP1 (1 µg of the protein). The binding reactions were performed with 1 µl (lane 1) and 2 µl (lane 2) of the template; G, G+A, C+T, and C are the corresponding Maxam–Gilbert sequencing reactions. Vertical bars and numbers indicate footprints. (E) footprinting analysis of the second conserved region and the adjacent regions.

Nine protected regions on the coding DNA strand (Fig. 3A, B) and six protected regions on the non-coding strand (Fig. 3C) were found in the first region [−267/+54 bp]. Potential binding sites for transcription factors YY1, TBP, SP1, AP2, NFY, Oct1, and many others were revealed by the MatInspector and Match™ software (Fig. S2, Table S1). The presence of these potential binding sites in the promoter region is in agreement with the results of competitive inhibition of EMSA (electrophorectic mobility shift assay) with oligonucleotides containing known binding sites for TBP («Promega»), YY1 (SRE-element) [23], Sp1 («Promega»), and CBF (CAAT-binding factor) [24] (Fig S4). The binding sites for TBP and YY1 were also found in the homologous regions of mouse and human [15], [17].

We carried out footprinting binding reactions with recombinant SP1. Subsequent treatment with DNase I decreased intensity of the bands and an additional band at the edge of the binding site could be observed (Fig. 3D). Note this DNA motif almost completely corresponded to the protected region V [−75/−55 bp] (coding strand) and the region (2) [−59/−54 bp] (non-coding strand) identified in the footprinting reactions with the nuclear extracts (Fig. 3A,B,C). Protected region IV [−53/−39 bp] overlapped with a potential binding site for the transcription factor AP2. EMSA performed with oligonucleotides containing AP2 site [−53/−36 bp] has shown that AP2 can interact with the vole *Xist* promoter *in vitro* (Fig. S5).

DNase I *in vitro* footprinting with the second region [−551/−372 bp] of the vole *Xist* promoter revealed nine protected motifs in the coding and five in the non-coding DNA strands (Fig. 3E). These motifs overlapped with potential binding sites for the transcription factors NMP4, RAR_RXR, SATB1, HMGIY, Znf217, ERα, etc. (Fig. S3, Table S1).

### Functional Analysis of the Vole *Xist* 5′ Regulatory Region

**Figure 4:**
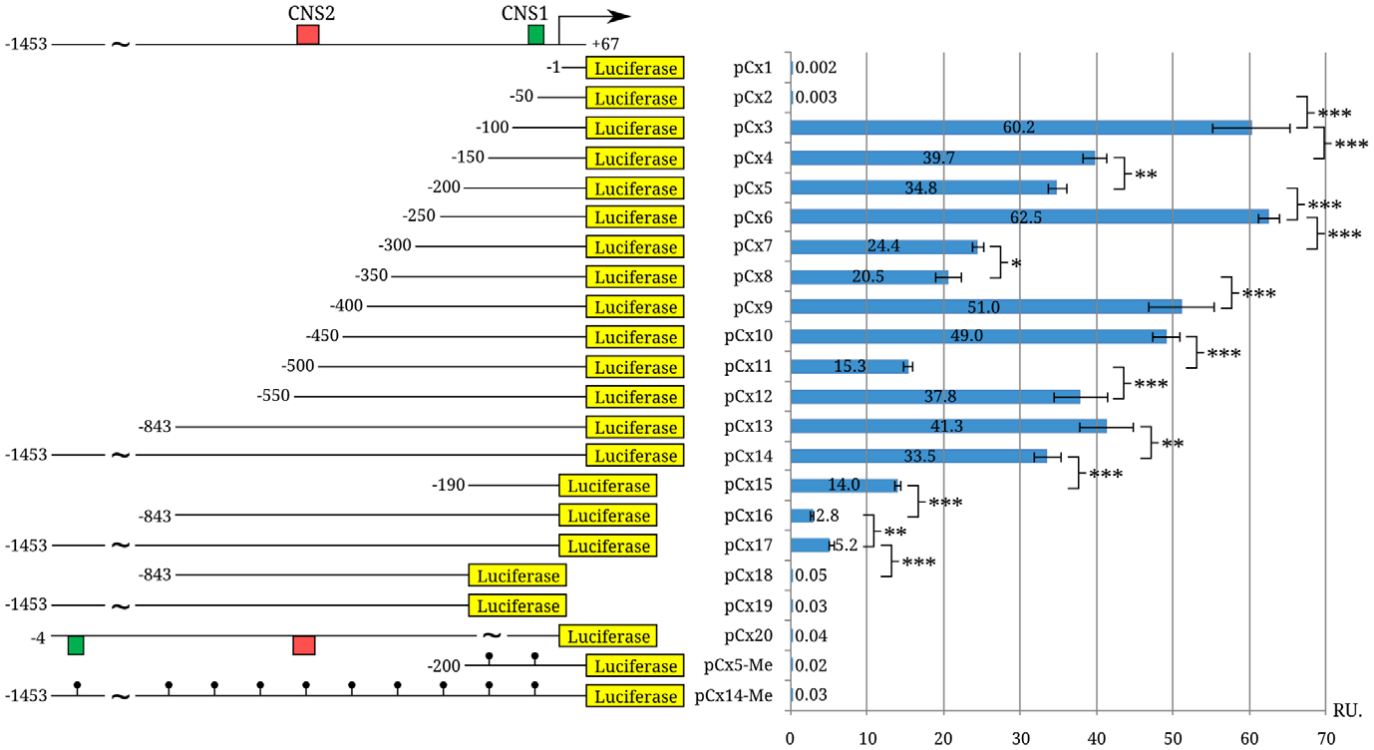
Analysis of activity of reporter constructs. Scheme of the *Xist* 5′ region is shown above; CNS1 and CNS2 are shown with green and red rectangles, respectively. pCx1–pCx14-Me are the variants of reporter constructs in the vector pGL4.10[luc2]; nucleotide positions relative to the Xist transcription start site are shown to the left. Diagram shows the relative luciferase activity of the constructs in female vole fibroblast culture (Sd10). Arrow and black dots indicate the *Xist* transcription start site and methylated CpG dinucleotides, respectively; R.U., relative luciferase activity units. Significant differences *** P≥0.999, ** P≥0.99, and * P≥0.95.

To determine influence of the potential regulatory elements on *Xist* expression we made 22 reporter constructs containing a luciferase gene under control of different fragments of the vole *Xist* promoter. The fragments overlapped the region [−1453/+67 bp] and differed from each other in 50 bp (Fig. 4). Female vole fibroblasts (Sd10 cell line) were used for transient transfection.

All the reporter constructs could be divided into two classes: the constructs contained the region [−4/+67 bp] (Fig. 4, pCx1–pCx14) and those with deletion of this region (pCx15–pCx17). A deletion of the region [−4/+67 bp] caused a significant decreasing in activity of the reporter constructs whereas a deletion of the region [−190/+67 bp] (pCx18 и pCx19) repressed completely the activity of the luciferase gene. The constructs pCx1 [+1/+67 bp] and pCx2 [−50/+67 bp] did not have any significant luciferase activity while the region [−100/−50 bp] in the consruct pCx3 [−100/+67 bp] provided the highest activity of the reporter gene. This construct comprised the protected region V binding SP1 and the region IV bearing potential AP2 binding site (Fig. 3A, D). Thus, one can conclude that the minimal promoter is localized at positions [−100/+67 bp] and SP1 is one of *Xist* transcription activators.

Among the constructs contained more than 100 bp of the vole *Xist* 5′ region, we found an increase in the reporter gene expression for the constructs pCx6 (by 27 units, P≥0.999), pCx9 (by 31 units, P≥0.999), and pCx12 (by 23 units, P≥0.999) and a decrease in the reporter gene activity for the constructs pCx4 (by 20 units, P≥0.99), pCx7 (by 38 units, P≥0.999), and pCx11 (by 34 units, P≥0.999) (Fig. 4). This suggests that the regions −100/−150, −250/−300, and −450/−500 bp can comprise negative regulatory elements, whereas the regions −200/−250, −350/−400, and −500/−550 bp can contain the elements activating *Xist* transcription. Decreasing in luciferase activity for the construct pCx14 [−1453/−843 bp] can be explained by the presence of CTCF binding site in CNS3 [−964/−944 bp] (Fig. 2).

The construct pCx20 comprising the region [−1453/−4 bp] in an inverse orientation failed to demonstrate any significant luciferase activity.

Methylation of CpG dinucleotides in gene promoters is known to be one of the epigenetic mechanisms involved in transcription regulation [25]. To understand the role of CpG methylation in *Xist* expression we treated the constructs pCx5 and pCx14 with M.Sss I which methylates cytosines in 5′-CG-3′ sequences. The luciferase activity of the methylated constructs (pCx5-Me and pCx14-Me) was assessed 48 h after their transient transfection into female vole fibroblasts (Sd10). Both constructs failed to display any significant luciferase activity (Fig. 4), thereby suggesting that CpG methylation completely blocks *Xist* transcription. This may be due to a suppression of binding of the methyl-sensitive transcription factors to the regulatory elements.

### Functional Analysis of the Vole *Xist* Interspecific −43G/A Substitution in minimal promoter Region

A single-nucleotide substitution of guanine (G) with adenine (A) at position −43 bp was found in the *M. arvalis* minimal promoter whereas the other vole species - *M. rossiaemeridionalis*, *M. transcaspicus*, and *M. kirgisorum* - contain G at this position. This substitution leads to a loss of one CpG dinucleotide in the *Xist* minimal promoter of *M. arvalis*. Therefore, we assumed that −43G/A substitution could reduce transcription of the *M. arvalis Xist* via increasing or decreasing the binding efficiency of a transcription factor, thus, providing skewed X-inactivation in *M. arvalis*×*M. rossiaemeridionalis* female hybrids. The binding site for the transcription factor CTCF was previously localized in the mouse and human *Xist* minimal promoter. The substitution of C with A at position −43 bp in this site repressed the human *XIST* expression while the substitution of C with G caused increased expression and skewed X-inactivation. The same correlation was found when studying the mouse *Xist* [14].

**Figure 5:**
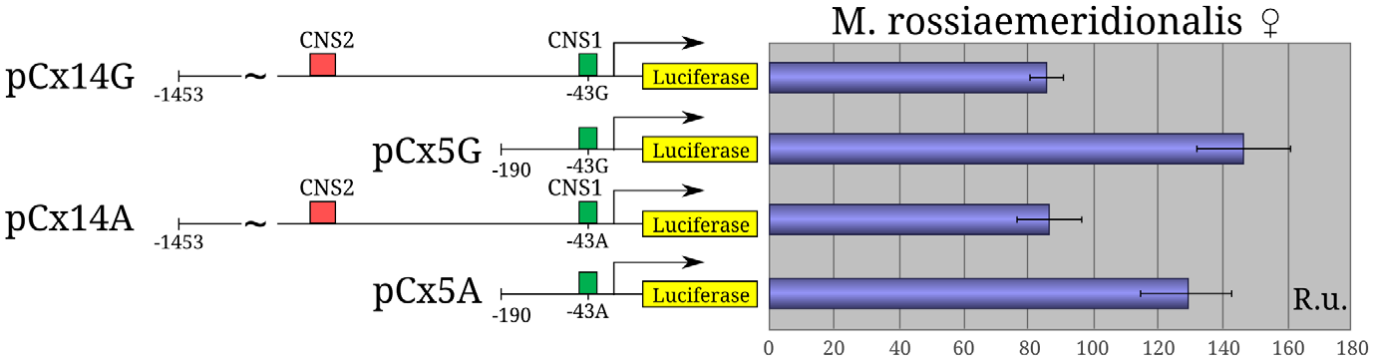
Analysis of the effect of −43G/A substitution on the activity of reporter constructs. Schemes of the constructs pCx14G/A and pCx5G/A are shown to the left; green and red rectangles denote the conserved regions CNS1 and CNS2, respectively. Relative luciferase activity of constructs in the fibroblast culture Sd10 is shown to the right. Arrow shows the *Xist* transcription start site; R.U., relative luciferase activity units.

To determine the functional significance of the substitution the constructs pCx14 G/A [−1453/+67 bp] and pCx5G/A [−190/+67 bp] bearing G or A at position −43 bp were used. Their activity was analyzed in the primary fibroblasts of *M. rossiaemeridionalis* and *M. arvalis* males and females as well as in primary female mouse fibroblasts. The data on the *M. rossiaemeridionalis* female fibroblasts (Sd10) are given in Fig. 5. We observed that the substitution −43G/A had no effect on the activity of the reporter constructs. Analogous results were obtained when using the other fibroblast cultures (data not shown).

In the region [−52/−40 bp], computational analysis of the vole *Xist* promoter revealed a DNA motif with a weak similarity to the known CTCF consensus (Fig. 2B). This region was located in CNS1 and corresponded to the protected motif IV (Fig. 3A). Using chromatin immunoprecipitation (ChIP) we have also shown that CTCF interacts *in vivo* with the *Xist* promoter region in vole *M. rossiaemeridionalis*.

**Figure 6:**
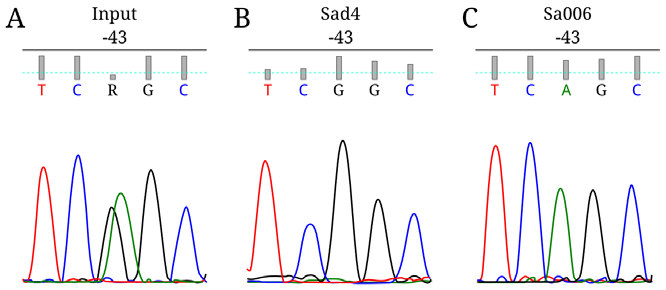
CTCF interacts with the *Xist* allele on the active X chromosome in voles. Sequencing of the vole *Xist* minimal promoter using as a template Sad4 or Sa006 genomic DNA (A), CTCF-bound fraction from line Sad4 (B), CTCF-bound fraction from line Sa006 (C).

CTCF interaction with *Xist* on the active or inactive X chromosome was studied by ChIP in two hybrid fibroblast lines, Sa006 and Sad4, obtained by subcloning of primary lung fibroblasts of *M. arvalis*×*M. rossiaemeridionalis* female hybrids. It has been previously demonstrated that the *M. rossiaemeridionalis* X chromosome is inactive in Sa006 cell culture and *M. arvalis* X chromosome is inactive in Sad4 cell culture [26]. Sequencing PCR products from CTCF bound fractions for both Sa006 and Sad4 cell lines detected only the *Xist* allele corresponded to the active X chromosome (the X chromosome of *M. arvalis* in Sa006 line and that of *M. rossiaemeridionalis* in Sad4 line) (Fig. 6). Thus, CTCF binds to the promoter of the transcriptionally inactive *Xist* allele in the vole fibroblasts.

CTCF is well conserved in mammals with 99% protein homology. We compiled vole mRNA and CTCF protein sequences based on the *Microtus ochrogaster* (closely related vole species) transcriptome published in databases (SRP002127). A comparison of the vole and human CTCF by FASTA has demonstrated 98.2% identity and 99.2% similarity at the amino acid level. In addition, Western-blot confirmed that the antibodies for the human CTCF (Upstate #07-729, Cell Signaling #2899) recognized a protein of 140 kDa in protein extracts from both human and vole fibroblasts (Fig. S6). Double-stranded oligonucleotides V-1 [−61/−17 bp] and V-II [−49/−5 bp] contained a potential CTCF binding site were used as probes. We also used DNA probe Pmin comprised the complete sequence of minimal promoter [−111/+27 bp] and a double-stranded oligonucleotide contained well-characterized CTCF binding site (F-II) from the insulator of chicken β-globin gene cluster [27] (positive control). CTCF formed a specific complex only with the control DNA probe (F-II) and no specific complexes were formed in the case of different regions of the vole promoter (V-I and V-II) and the entire minimal promoter (Pmin) (Fig. S7).

**Figure 7:**
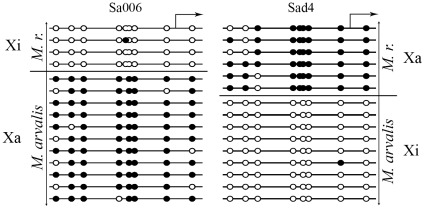
Methylation profile of the *Xist* 5′ regulatory region in the Sa006 and Sad4 vole fibroblast lines. White circles denote unmethylated CpG sites; black, methylated; the *Xist* transcription start site is indicated by arrow; Xa, active X chromosome; Xi, inactive X chromosome; *M. r.*, *Microtus rossiaemeridionalis*.

CTCF binding is known to be almost completely inhibited by CpG methylation [28]. Therefore, we investigated CpG methylation of the *Xist* minimal promoter region [−100/+5 bp] in the Sa006 and Sad4 cell lines (Fig. 7). The *M. rossiaemeridionalis* and *M. arvalis* alleles could be distinguished by the −43G/A substitution. In both cell lines, we observed that the inactive *Xist* allele (the active X chromosome) was hypermethylated (90±3% and 87±5% of the CpG dinucleotides analyzed for Sa006 and Sad4, respectively). At the same time the active *Xist* allele (the inactive X chromosome) was hypomethylated (3±3% and 1±1% for Sa006 and Sad4, respectively). Thus, the results of EMSA and CpG methylation assays imply that CTCF can not bind directly to the *Xist* minimal promoter on the active vole X-chromosome.

**Figure 8:**
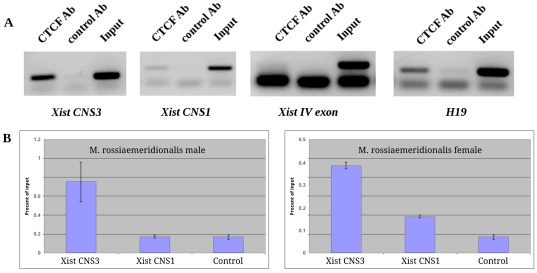
CTCF interaction with the vole *Xist* regulatory region. (A) ChIP performed using anti-CTCF monoclonal antibodies (CTCF Ab) followed by PCR analysis. The fourth *Xist* exon and Igf2/H19 ICR were used as negative and positive controls of CTCF binding, respectively. (B) Real-time PCR analysis of ChIP experiments. Each bar indicates the average of two independent PCR analyses with the standard deviation. Percent of input is calculated as described in Materials and Methods.

The third conserved region (CNS3) [−964/−944 bp] also comprises a potential CTCF binding site (Fig. 2A). When analyzed the data on CTCF ChIP-seq (http://genome.ucsc.edu.) in tissues and cell cultures of different mouse lines, we found CTCF binding to the mouse *Xist* promoter around [−1026/−1006 bp], corresponding to CNS3 (Fig. S8, Fig. 2A). We carried out ChIP with CTCF antibodies using chromatin isolated from male and female primary embryonic fibroblasts of *M. rossiaemeridionalis*. RealTime PCR with primers specific for CNS1 and CNS3 has shown that CTCF binds to the CNS3 bp region of the vole *Xist* promoter (6-fold enrichment in comparison with a negative control) (Fig. 8). Taking into account size variability of sonicated chromatin in ChIP reactions [29], [30], [31], we believe that CTCF binding to CNS3 could be detected in ChIP experiments using the primers for CNS1.

## Discussion

X-inactivation occurs in early embryogenesis of female mammals, before initiation of X-inactivation, *Xist* is expressed on both X chromosomes at a low level. During the initiation stage the *Xist* allele on the future inactive X chromosome is up-regulated whereas on the active X chromosome it is repressed. Thus, the *Xist* promoter has to comprise some elements activating *Xist* transcription and those responsible for its silencing in each female somatic cell. In this work, we attempted to shed more light on general principles of *Xist* expression regulation. We indentified the most conserved regions of the *Xist* promoter between vole and other mammalian species and characterized them by DNase I footprinting, luciferase reporter analysis, EMSA, and methylation assay.

The *Xist* minimal promoter spans the region [−100/+67 bp] and contains INR and binding sites for the transcription factors YY1, TBP, SP1, and AP2. In the EMSA experiments, the oligonucleotides with TBP («Promega») и YY1 (SRE-element) [23] binding sites successfully competed with the promoter region [−31/+19 bp] for binding proteins of vole liver nuclear extracts (Fig. S4). Using reporter constructs has shown that the elements localized in the region [−50/+67 bp] are necessary but not sufficient to activate the reporter gene. The minimal vole *Xist* promoter contributes significantly to increasing luciferase activity. It comprises CNS1 [−73/−44 bp] which overlaps the protected regions V, IV, and 2 indentified in the footprinting experiments with nuclear extract from Sd10 cells and Sp1 protein. The oligonucleotides contained Sp1 («Promega») binding site successfully competed with the promoter region [−95/−50 bp] in the EMSA experiments (Fig. S4). The conservatism of CNS1 [−73/−44 bp] seems to be due to the presence of the Sp1 binding site which was found in all the species analyzed (Fig. S1, S2) and studied in the mouse and human *Xist* promoters [15], [17]. This region is likely to be involved in *Xist* transcription activation on the inactive X chromosome in female somatic cells.

In addition to the SP1 binding site, the protected regions V and 2 contain a potential binding site for the transcription factor BTEB3/KLF13. Unlike SP1, BTEB3/KLF13 is a repressor and can compete with SP1 for the binding site [32]. Therefore, BTEB3/KLF13 may interact with the *Xist* promoter on the active X chromosome and inhibit its expression.

The protected region IV [−53/−40 bp] overlaps the potential AP2 binding site (Fig. S2). According to EMSA, AP2 can bind to this region *in vitro* (Fig. S5). Both AP2 and SP1 activate transcription and appear to be strong activators of vole Xist expression. Moreover, close location of AP2 and SP1 binding sites suggests their cooperation [33]. The AP2 binding site is species-specific and is revealed by MatInspector only in the vole *Xist* promoter but the −43G/A substitution disrupts this site in *M. arvalis*. This part of the *Xist* promoter region is well conserved in different mammalian species and contains A at position −43 (Fig. S1, Fig. 2B). One can speculate that binding the additional AP2 activator to the *Xist* allele bearing G at position −43 may cause skewing choice of the inactive X chromosome in female vole hybrids between *M. arvalis* and other closely related species. However, analysis of reporter constructs containing A or G at position −43 bp in male and female somatic cells has not revealed any significant difference in their luciferase activity. Thus, skewed choice of the inactive X chromosome in vole interspecific female hybrids seems to be a more complicated phenomenon.

A CTCF binding site was experimentally found in the mouse and human region homologous to the *Xist* promoter region containing AP2 binding site in *M. rossiaemeridionalis*. CTCF has been shown to be a transcription activator and interact with the *Xist* allele on the inactive X chromosome. Interestingly, a single nucleotide C/A substitution at position −40 bp in mouse and −43 bp in human has led to a dramatic decrease in CTCF binding both *in vitro* and *in vivo*. A C/G substitution at the same positions cased an increase in binding and involvement of additional CTCF zinc fingers. In both mouse and human, the substitutions resulted in skewed choice of the inactive X chromosome favoring a more active *Xist* allele [14]. A multiple alignment of the minimal promoter of several mammals has demonstrated that the CTCF binding region revealed by Pugacheva et al [14] in the human *Xist* is not well conserved (Fig. S1, Fig. 2B). However, analysis of the *Xist* minimal promoter by CTCFBS Prediction Tool in CTCFBSDB database (a CTCF binding site database, http://insulatordb.uthsc.edu) found these CTCF binding sites in all the species studied except for rat and vole. In addition, we were not able to confirm CTCF binding with the regions V-1 [−61/−17 bp] and V-II [−49/−5 bp] by EMSA in vole. The presence of CTCF binding site in the minimal promoter is likely to be a species-specific peculiarity of *Xist* transcription regulation in rodents.

This study has shown CTCF binding to the inactive *Xist* allele in vole female hybrids and males using ChIP. A multiple alignment of *Xist* 5′ region of different species revealed a 25 bp region (CNS3) which is conserved and comprises a potential CTCF binding site. ChIP-seq data published on UCSC Genome Browser have demonstrated that a strong peak of CTCF binding is observed at positions −1020 bp and −3000 bp relatively *Xist* transcription start site in mouse and human, respectively (Fig. S8, S9). This binding corresponds to the CTCF binding site from CNS3 (Fig. 2B). As the minimal promoter of the inactive *Xist* allele is hypermethylated in vole, its interaction with CTCF must be inhibited [28]. This allows us to suggest that the potential CTCF binding site from CNS3 was analyzed in our ChIP experiments.

The second strong peak of CTCF binding to the mouse and human *Xist* is localized in the first exon. In mouse ChIP-seq experiments, CTCF binding was observed in all cases for the first exon and in 40% (7 out of 17) of cases for the promoter region (Fig. S8). In human, both CTCF binding peaks were detected in each experiment irrespective of sex, tissue or cell culture (Fig. S9). Two active binding sites flanking *Xist* promoter and first exon may imply that CTCF is involved in formation of an inactive chromatin domain on the active X chromosome in males and females, leading to a transcription repression of this *Xist* allele.

According to the reporter constructs analysis, the vole region [−150/−100 bp] may contain some negative regulatory elements for *Xist* transcription (Fig. 4). This region corresponds to the protected motifs 4, VII/5, and VIII/6 which potentially interact with NFY, Oct1, and some other homeodomain-containing transcription factors (Fig. S2, Table S1). NFY can repress transcription directly or via chromatin modification in the *Xist* promoter on the active X chromosome [34]. Among all the homeodomain-containing transcription factors, Msx1 (Msh homeobox 1) is of the greatest interest because it has three potential binding sites in this region. Msx1 can interact with other transcription factors, components of the transcription complex and acts as a repressor during embryogenesis. However, it is currently unclear which of its sites is functionally important or whether they act jointly.

The protected region IX is localized in the region [−200/−150 bp]. It comprises a potential binding site for the transcription factor TCF11/MAFG (Fig. S2, Table S1). The presence of this region in the reporter construct pCx5 slightly decreased its activity in comparison with pCx4, suggesting that TCF11/MAFG may be a repressor of *Xist* transcription (Fig. 4).

An increase in the activity of pCx6 containing the region [−250/+67 bp] is unclear, since this region has not been analyzed by footprinting. However, multiple alignment revealed a high conserved region which overlaps completely with a potential binding site for Jarid2 playing a key role in development and embryonic stem cell differentiation (Fig. S1). This factor has been shown to be a component of PRC2 (Polycomb repressive complex 2), which mediates histone H3 ‘Lys-27’ trimethylation (H3K27me3) [35], [36]. It is well known that X-inactivation occurs in early embryogenesis and PRC2 complex is involved in the process [1]–[3]. Moreover, H3K27me3 participates in X-linked gene silencing during X-inactivation and *Xist* repression on the active X chromosome. Thus, this is in agreement with Jarid2 functions and could explain the conservatism of Jarid2 binding site in *Xist* promoter of the species analyzed.

pCx7 [−300/+67 bp] activity is significantly decreased (Fig. 4). The data on the mouse *Xist* regulatory region [15], [16] suggest localization of a repressor in the region [−300/−250 bp] of the vole *Xist* promoter. However, this region it is not conserved and has not been analyzed by footprinting so a potential factor involved is unknown. pCx8 demonstrates almost the same luciferase activity. A moderately conserved 30 bp region was revealed in the region [−350/−300 bp] by multiple alignment of *Xist* promoters of 13 mammalian species using Vista (Fig. S1). It contains a potential Oct1 binding site which can recruit additional factors, including repressors [37].

Additional regulatory elements were found upstream of the minimal promoter in the region [−550/−350 bp] containing CNS2. As shown by reporter construct assay, they can both activate and repress *Xist* transcription in voles. 14 motifs protected from DNase I have been detected in CNS2 and the adjacent sequences (Fig. 3E). All the potential binding sites indentified in this region are summarized in Table S1. The most important sites will be discussed below.

Increasing pCx9 [−400/+67 bp] luciferase activity agrees with the presence of the protected motifs 4, 5 and I. They correspond to potential binding sites for transcription factors NMP4, NFAT, and RAR_RXR (Fig. S3). RAR_RXR is a heterodimer of retinoid receptors which can recruit histone acetyltransferases and the protein HMGA1 [38], leading to formation of an “open” chromatin structure and gene expression activation. In the presence of retinoic acid RAR_RXR is also able to recruit transcription factors and RNA polymerase II [39]. Retinoids are regulators responsible for embryonic morphogenesis and differentiation of many tissues during post-embryonic development (epithelial and hematopoietic cells). Thus, retinoids may activate *Xist* expression on the future inactive X chromosome in early embryogenesis.

The level of pCx10 [−450/+67 bp] activity is comparable to that of pCx9. The region [−450/−400 bp] comprises the protected motifs 3, 4, II, III, and IV. The first three motifs overlap with the potential SRY binding site. SRY may regulate *Xist* expression only in males. However, the nuclear protein extract from female fibroblasts, where *SRY* is not expressed, was used in the footprinting reactions. Therefore, some other regulatory factor can bind to the motifs 3, 4, and II in females. The fact that the activity of the reporter constructs containing the 5′ region of mouse *Xist* did not differ between XX and XY cell lines [16] also confirms this conclusion. The protected motif IV partially overlaps with potential binding sites for ERα, NFY, and SATB1 (Fig. S3). SATB1 belongs to architectural proteins and interacts with S/MARs (Scaffold/Matrix Attachment Regions). It can also recruit co-repressors (HDAC), co-activators (HAT), and components of chromatin remodeling complexes [40], [41]. SATB1 binding sites were observed in the *Xist* 5′ region of all the mammalian species analyzed, although they are located at different positions relative to the *Xist* transcription start site. Its expression is detected in embryogenesis and embryonic stem cells. During X-inactivation, SATB1 is involved in formation of loop domains and compartmentalization of the inactive X chromosome in the nucleus. *Xist* is located in such a domain [42]. In an adult organism, *SATB1* is expressed only in the T- and B-lymphocyte precursors [43]. SATB1 recently demonstrated to be necessary for *Xist* RNA–dependent silencing in tumor T cells [44].

Repression of pCx11 luciferase activity is connected with addition of the region [−500/−450 bp] containing the protected motifs V, VI, and 2. The vole region [−480/−450 bp] is quite conserved in the species analyzed (Fig. S1). The transcription factors Znf217, RAR_RXR and receptors ERα, GRE, AR, RORA2, ESRRB can bind to these motifs (Fig. S3). Znf217 interacts with the co-repressor CtBP (C-terminal Binding Protein) and inhibits expression of target genes [45]. ERα and ERRB are estrogen receptors expressed beginning at the blastocyst stage. Since random X-inactivation takes place approximately at the implantation stage [46], estrogen binding to the receptors may directly or indirectly regulate expression of *Xist*. In addition, E3 ubiquitin ligase Rnf12 being an activator of *Xist* expression is an ERα cofactor and apparently elevates the transcription level of estrogen-sensitive genes [47], [48], [49]. Therefore, *Xist* transcription during X-inactivation at the implantation stage may be activated by Rnf12 and ERα.

pCx12 [−550/−500 bp] comprises CNS2 [−540/−498 bp] and its luciferase activity is 2.5-fold higher than that of pCx11. This appears to be related to interaction of this region with the transcription factors STAT3 and HMGIY (HMGA1). Potential binding sites for STAT3 and HMGA1 overlap with motifs 1 and VII (Fig. S3). The potential HMGA1 binding site was found in all species analyzed. HMGA1 regulates expression of various genes by modifying chromatin structure and recruiting other transcription factors via protein–protein interactions. HMGA1 is expressed in embryonic fibroblasts and in several cell types (bone marrow cells, macrophages, tumor cells) of adults [50]. In embryogenesis, HMGA1 may interact with *Xist* 5′ region on the future inactive X chromosome, leading to “open” chromatin structure and activation of *Xist* expression. No potential transcription factor binding sites were observed in the protected motifs VIII and IX. Some other yet uncharacterized regulatory proteins may be discovered in this region.

The constructs pCx13 and pCx14 containing the regions [−843/+67 bp] and [−1453/+67 bp], respectively, demonstrated the same luciferase activity as pCx12 [−550/+67 bp] (Fig. 4), implying that there is no elements influencing transcription of vole *Xist* upstream of position −550 bp. pCx14 has a lower level of luciferase activity than pCx13 and contains a CTCF binding site. However, a 600 bp difference in size between constructions does not allow us to make any conclusions about repressive role of CTCF based on reporter construct assay.

Thus, *Xist* expression in vole is regulated by numerous regulatory elements. The core promoter bears weak TATA box and initiator element and is involved in recruiting, correct orientation, and assembly of the preinitiation complex. These elements are necessary but not sufficient to initiate transcription at a high level. Therefore, additional elements of promoter (transcription factors SP1, AP2, and others) may be involved in activation and fine-tuning of *Xist* transcription. Since the status of *Xist* allele and whole X chromosome is established at the implantation stage, we paid close attention to potential binding sites for factors acting in early embryonic development. For example, a potential binding site for Jarid2 which recruits PRC2 in embryonic stem cells and participates in gene silencing was observed in the *Xist* promoter. The regulatory factors RAR_RXR, AR, GR, RORA, and ERα/Rnf12 can take part in *Xist* activation during initiation of X-inactivation in undifferentiated cells. HMGA1 and SATB1 belonging to architectural proteins appear to form specific chromatin structures on the future active and inactive X chromosomes, providing a platform for binding the corresponding transcription factors.

*Xist* expression can be regulated not only by transcription factors but also by CpG methylation of the promoter region. The function of such CpG methylation is to provide active state of one X chromosome by repressing *Xist* expression. Indeed, vole *Xist* minimal promoter is completely methylated on the active X chromosome and not methylated on the inactive one (Fig. 7). Moreover, study of luciferase activity of methylated reporter constructs (pCx5-Me and pCx14-Me) has shown that methylation of 5′ region effectively represses *Xist* expression (Fig. 4).

Surprisingly, in the vole *Xist* minimal promoter we did not find the CTCF binding site described in human and mouse. Instead, CTCF binding was revealed in the 5′ flanking region of the *Xist* promoter on the active X chromosome in both males and females. This site is conserved in different mammalian species. We believe that CTCF is an insulator which defines an inactive *Xist* domain on the active X chromosome in voles.

## Materials and Methods

### Ethics statement

The study was carried out according to “The Guidelines for Manipulations with Experimental Animals.” The study was approved by the Ethical Committee of the Institute of Cytology and Genetics, Novosibirsk, permit number: (order of the Presidium of the Russian Academy of Sciences of April 02, 1980 no. 12000-496).

### Computational Analysis of Nucleotide Sequences

The nucleotide sequences of *Xist* 5′ region of different mammalian lineages were extracted from the GenBank. They were analyzed using the mVista (http://genome.lbl.gov/vista/mvista/submit.shtml) software [51] (the parameters used for constructing plot: Calc Window, 20 bp and Min Cons Width, 20 bp) and CLUSTALW2 (http://www.ebi.ac.uk/Tools/clustalw2/index.html) [52]. The potential transcription factor binding sites were searched for using the programs MatInspector (http://www.genomatix.de) [53] and Match™ (http://www.gene-regulation.com) [54].

### Cell Cultures

Primary lung and embryonic fibroblasts of *M. rossiaemeridionalis* male (MsEf3♂) and female (Sd10♀), *M. arvalis* male (Maf♂) and female (Maf2♀) and primary female mouse fibroblasts (EFM2♀) were derived as previously described [55], [56]. Lines Sa006 and Sad4 were obtained by subcloning of the primary lung fibroblast cultures of hybrid females *M. rossiaemeridionalis* ♀×*M. arvalis* ♂. In all Sa006 cells, the *M. rossiaemeridionalis* X chromosome is inactive while that of *M. arvalis* is inactive in Sad4 line [26].

### Preparation of Nuclear Extracts and DNase I *in vitro* Footprinting

Nuclear extracts from liver cells and fibroblasts (line sd10) of *M. rossiaemeridionalis* were isolated as described [57], [58] and using a CelLytic™ NuCLEAR™ Extraction kit (Sigma). The nuclear extracts were stored at −70°C.

Two DNA fragments corresponding to the regions [−267/+54 bp] (Pmin) and [−551/−372 bp] were used as probes. These probes were amplified by PCR with the primer pairs Msx7 5′-tatgtggcctttcctataagc-3′/Supr7 5′-tagatggacagagaccacagagg-3′ and ERR 5′-atagcccctcgtcttggtgac-3′/ERF 5′-cgctgagctgtttctctaccg-3′, respectively. Before PCR one primer was labeled at the 5′ end with [γ^_32^P]ATP and T4 polynucleotide kinase (Promega). The binding reactions were carried out according to the protocol recommended for Core Footprinting System (Promega). Reaction mixtures were supplemented with 5–50 µg of the nuclear protein extract or 2 µg of the recombinant transcription factor SP1. Nonspecific protein binding was inhibited by adding 0.1–1.0 µg of Poly(dI-dC) • Poly(dI-dC) (Sigma). To determine the nucleotide sequences of the bound regions Maxam–Gilbert sequencing reactions were performed concurrently with the footprinting. The samples were analyzed in a 4–6% denaturing polyacrylamide gel.

### Electrophoretic Mobility Shift Assay

Double-stranded oligonucleotides (probes F-II, V-I, V-II, AP2K, AP2-43G) and PCR product (probe Pmin) were radioactively labeled and used in EMSA. The double-stranded oligonucleotides were obtained by annealing complementary single-stranded oligonucleotides and were labeled with [γ-^32^P]ATP and T4 polynucleotide kinase (Promega). The single-stranded oligonucleotides are given in Table S2.

To analyze CTCF binding, 3 µl of 5× buffer (125 mM HEPES pH 7.9, 250 mM KCl, 31.25 mM MgCl_2_, 5% Nonidet P-40, and 25% glycerin), 2 pmol of DNA probe, 300 ng of recombinant CTCF protein (Novus Biologicals) were mixed and incubated for 30 min at a room temperature. When studied AP2 binding, 1 µl of buffer (20% glycerin, 5 mM MgCl_2_, 2.5 mM EDTA, 2.5 mM DTT, 250 mM NaCl, and 50 mM Tris–HCl pH 7.5), 2 pmol of DNA probe, and 1 µl of AP2 extract (Core Footprinting System kit, Promega) were mixed and incubated for 10 min at a room temperature. After the incubation, the samples were analyzed in 6% polyacrylamide gel (AA/bisAA, 29∶1) in 1× TBE buffer. The electrophoresis was carried out at a voltage of 10 V/cm. The dried gel was exposed with X-ray film for 12–24 h.

### Chromatin Immunoprecipitation (ChIP)

Vole fibroblasts (Sd10, Sa006, and Sad4 lines) were cultivated as previously described [26], [56]. Approximately 10^7^ cells were used. Chromatin was cross-linked *in situ* by incubating the cells in 1% formaldehyde for 10 min at 37°C. The DNA–protein complexes were immunoprecipitated with anti-CTCF antibody (Upstate #07-729, Cell Signaling #2899) and nProtein A Sepharose 4 Fast Flow beads (Amersham Biosciences) according to the protocol for Chromatin Immunoprecipitation (ChIP) Assay Kit (Upstate).

CTCF bound fraction was amplified by PCR with primers spanning the *Xist* minimal promoter (sense primer 5′-tatgtgcctttcctataagc-3′ and antisense primer 5′-tcccagagaccccgatagatg-3′) and the region around −940 bp (sense primer 5′-tgacaagacatgggtttcttgaggcg-3′ and antisense primer 5′-tgaacatcgcagtggttcacataggg-3′). The PCR conditions were 95°C for 5 min and 30 cycles of 95°C for 30 s, 60°C for 30 s, and 72°C for 40 s. A CTCF binding site within the vole Igf2/H19 ICR (sense primer 5′-agagaccacagaggaggctcacatc-3′ and antisense primer 5′-aggtgcaagggtaccactctagagg-3′) was used as a positive control. The PCR conditions were 95°C for 2 min and 30 cycles of 95°C for 30 s, 62°C for 40 s, and 72°C for 40 s. PCR products of CTCF bound fractions from the Sa006 and Sad4 cell lines were sequenced. The *M. rossiaemeridionalis* and *M. arvalis* alleles were distinguished by G/A polymorphism at position −43 bp.

### Real-time PCR

Quantitation of DNA in ChIP bound fraction was performed by real-time PCR using the Bio-Rad iQ5 real-time PCR detection system. PCR products were detected with SYBR Green. Real-time PCR was carried out in duplicate on control, ChIP, and input DNA samples at the following thermal cycling parameters 95°C for 5 min and 40 cycles of 95°C for 10 s, 58°C for 15 s, and 72°C for 25 s. Data were collected at 78°C for primer pairs 5′-tgacaagacatgggtttcttgaggcg-3′ and 5′-tgaacatcgcagtggttcacataggg-3′ (CNS3, the region around −940 bp), 5′-gagctgagatgggacaattctc-3′ and 5′-tacttctggggaaagatctgga-3′ (the fourth *Xist* exon, negative control) and at 84°C for primer pairs 5′-tatgtgcctttcctataagc-3′ and 5′-tcccagagaccccgatagatg-3′ (*Xist* minimal promoter). Data were analyzed by ΔC_T_ method as described in Imprint chromatin immunoprecipitation kit protocol (Sigma; Cat. no. CHP1) and presented as percentage of input chromatin. We used next equation %Input = 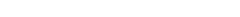, input dilution factor is 20 (5% of total chromatin were used as input).

### Western blotting

Total cell lysates were prepared directly in SDS-PAGE loading buffer. Western blot analysis was performed after electrophoretic separation of polypeptides by 10% SDS–PAGE and transfer to nitrocellulose membrane (Bio-Rad). Blots were probed with the primary anti-CTCF and appropriate secondary antibodies, and detected by chemiluminescence with 1.25 mM luminol (Sigma; Cat. no. A-8511) in 0.1 M Tris-HCl pH 8.5, 68 mM p-Coumaric acid (Sigma; Cat. no. C-9008) and 33% hydrogene peroxide. Antibodies were used at the following dilutions: primary anti-CTCF (Upstate #07-729, Cell Signaling #2899) 1∶1000, secondary anti-rabbit IgG peroxidase conjugated (Sigma; Cat. no. A-0545) 1∶80000.

### Transient Transfection and Measurement of Luciferase Activity

Fragments of vole *Xist* promoter were amplified by PCR and cloned into the vector pGL4.10[luc2] (Promega). The nucleotide sequence of the insert was verified by sequencing. The cells were transfected using Lipofectamine™2000 (Invitrogen). The fibroblast cell line of female *M. rossiaemeridionalis* was concurrently transfected with the reporter and control (pGL-4.74[hRluc/TK], Promega) plasmids at a ratio of 10∶1. Luciferase activity was determined 48 h after transfection by a Dual-Luciferase® Reporter Assay System (Promega) according to the manufacturer's protocol. The luciferase activity was recorded in a Wallac 1420 multilabel counter using the following parameters: measurement time, 1 s; and delay duration, 0.1 s. The ratio of Firefly to *Renilla* luciferase activities was taken as the activity of reporter construct. The activity of each reporter construct was measured in three independent experiments. The significance of differences in the reporter gene expression was estimated using Fisher's test.

### Methylation of Cytosine Residues in Plasmid DNA

The cytosine residues in reporter constructs were methylated with M.SssI CpG methyltransferase (NEB). The components of reaction mixture - 12 µl S-adenosylmethionine (0.16 mM, NEB), 12 µl of 10× NEB buffer 2, 24 U of M.SssI methyltransferase (NEB), and 6 µg of plasmid DNA - were mixed and the volume was adjusted to 120 µl with bidistilled water. The reaction components were mixed and incubated for 1 h at 37°C. The reaction was stopped by heating for 20 min at 65°C. DNA was extracted with phenol–chloroform and precipitated with ethanol.

### Bisulfite Sequencing

Genomic DNA (2 µg) was treated with bisulfite according to the manufacturer's protocol (EpiTect Bisulfite kit, Qiagen) and eluted in 20 µl of elution buffer. The PCR product was amplified by nested PCR using the bisulfite-converted DNA as a template. The primers specific for the modified DNA (forward primer 5′-tttgttatgagttttggtataatta-3′ and reverse primer 5′-aaaaactaaaaatattcccaaaaac-3′) were used in the first PCR round. The following PCR conditions were used: 95°C for 5 min; 30 cycles of 95°C for 15 s, 54°C for 15 s, and 72°C for 20 s, and final extension at 72°C for 5 min. In the second round, the PCR product from the first round was used as a template and the primers confined a shorter fragment located inside the PCR product (forward primer 5′-tttaattattttttttagaaaatagtttgt-3′ and reverse primer 5′-aaaaaccacaaaaaaaactcacatc-3′). The reaction was carried out under the same conditions. The bisulfite PCR products were gel purified and cloned into pGEM-T Easy (Promega), and independent clones were sequenced. Sequence analysis was visualized using MethTools [59].

## Supporting Information

Figure S1 The alignment of 5′ regulatory regions and parts of the first exon of *Xist* in different mammalian lineages. (Bt) *Bos taurus*; (Cf) *Canis familiaris*; (Dn) *Dasypus novemcinctus* (armadillo); (Ec) *Equus caballus* (horse); (Hs) *Homo sapiens*; (La) *Loxodonta africana* (elephant); (Oc) O*ryctolagus cuniculus* (rabbit); (Sa) *Sorex araneus* (shrew); (Ss) *Sus scrofa* (pig), (Cp) *Cavia porcellus* (guinea pig), (Mm) *Mus musculus*; (Rn) *Rattus norvegicus*; (Mr) *M. rossiaemeridionalis* (vole). Conserved regions are shown with red frames; arrow indicates *Xist* transcription start site.
(PDF)

Figure S2 Computational analysis of the promoter region of the *M. rossiaemeridionalis Xist* gene. Consensuses of several identified potential transcription factor binding sites are shown above and below the nucleotide sequence. The sequence corresponding to the first conserved region, CNS1, is framed with a rectangle. Footprints are shown with red lines and numerals. Roman numerals denote the protected DNA motifs identified in the (+)-strand and Arabic numerals, in the (−)-strand; arrow shows the transcription start site. The nucleotides conserved for vole, human, cow, dog, horse, and rabbit are shown in yellow.
(TIF)

Figure S3 Computational analysis of the second conserved region (CNS2) and the adjacent sequences in the 5′ region of the *M. rossiaemeridionalis Xist* gene. Consensuses of several identified potential transcription factor binding sites are shown above and below the nucleotide sequence. The sequence corresponding to CNS2 is framed with a rectangle. Footprints are shown with red lines and numerals. Roman numerals denote the protected DNA motifs identified in the (+)-strand and Arabic numerals, in the (−)-strand. In CNS2, the nucleotides conserved for vole, human, cow, dog, horse, and rabbit are shown in yellow.
(TIF)

Figure S4 (A) Interaction of the vole *Xist* minimal promoter with the liver nuclear extract. (−) [γ-^32^P]ATP labeled DNA fragments (0.05 pmol) without incubation with the nuclear extract (NE). (+) [γ-^32^P]ATP labeled DNA fragments (0.05 pmol) incubated with NE (5–10 µg) (Buffer: 10 mM HEPES pH 7.6, 40 mM KCl, 2 mM MgCl_2_, 0.1 mM EDTA, 0.5 mM PMSF, 1 mM DTT, 10% glycerol). The regions [−31/+19 bp] and [−70/−25 bp] of the vole *Xist* promoter were obtained by hydrolysis of a PCR product with *Sau*3AI. The primers used were 5′-cacgggaaactggcaaacat-3′ and 5′-cactcctcttctggtctct-3′. The [−95/−50 bp] fragment was amplificated with 5′-gaagtcgggacttttccgc-3′ and 5′-agagaccagaagaggagtg-3′. The promoter region [−110/−78 bp] was amplificated with 5′-taaaacgccaataagaag-3′ and 5′-gcggaaaagtcccgacttc-3′ primers. The fragments [−155/−90 bp] and [−212/−155 bp] were generated by hydrolysis of a PCR product with *Vsp*I. The primers used were 5′-cccgacttcttattggcgtttta-3′ and 5′-atatacaaatttggtggttctc-3′. (B) Competitive inhibition of EMSA. (NE) [γ-^32^P]ATP labeled DNA fragments (0.05 pmol) with nuclear extract only. In the other reactions, unlabeled double-stranded oligonucleotides (5 pmol; 100x) containing binding sites for a number of transcription factors were added. (TBP) 5′-gcagagcatataaggtgaggtagga-3′ oligonucleotides with the TBP binding site («Promega»); (SRE) 5′-cagtacaggatgtccatattaggacacatctgcgt-3′ oligonucleotides with the YY1 binding SRE-element [23]; (SP1) 5′-attcgatcggggcggggcgagc-3′ oligonucleotides with the Sp1 binding site («Promega»); (CBF) 5′-cgtctccaccaatgggagggctggc-3′ oligonucleotides with the CBF binding site [24]. The fragment [−31/+19 bp] of the vole *Xist* promoter gave two retardation bands in EMSA. An addition of unlabeled the “TBP” oligonucleotides resulted in a loss of the band corresponding to a more electrophoretic mobile complex. When added the “SRE” oligonucleotides a less mobile complex disappeared. The fragment [−95/−50 bp] comprises a potential Sp1 binding site. In EMSA, it yielded three retardation bands. An addition of the “SP1” oligonucleotides led to a loss of high-molecular retardation bands, which is typical of the Sp1 factor. The region [−110/−78 bp] gave two retardation bands. Formation of a less mobile complex was inhibited with “CBF” oligonucleotides but not with those comprising the Sp1 binding site, suggesting this region binds CBF or another CAAT-binding factor, for example NFY detected by MatInspector.
(TIF)

Figure S5 AP2 binding to the vole *Xist* promoter *in vitro*. C, DNA probe containing consensus binding site for AP2 (positive control); AP2–43G, DNA probe containing the [−54/−36 bp] region with guanine at position −43 bp; (+) denotes the lanes with the DNA probes incubated with AP2 extract and (−) without AP2 extract. Arrow indicates the specific complex formed by AP2 binding to DNA probe.
(TIF)

Figure S6 Western blot analysis of extracts from vole and human fibroblasts using CTCF antibody - Cell Signaling #2899 (A), Upstate #07-729 (B).
(TIF)

Figure S7 EMSA analysis of the CTCF transcription factor binding to the *Xist* minimal promoter. (+) indicates the lanes with DNA probes incubated with CTCF and (−) without CTCF. Arrow denotes the specific complex formed by CTCF binding to F-II probe.
(TIF)

Figure S8 Chip-seq data on CTCF binding in mouse (UCSC genome browser). Significant sites are shown with rectangles. The *Xist* minimal promoter is indicated in yellow.
(TIFF)

Figure S9 Chip-seq data on CTCF binding in human (UCSC genome browser). Significant sites are shown with rectangles. The *Xist* minimal promoter is indicated in yellow.
(PDF)

Table S1 Description of the transcription factors with potential binding sites identified in the 5′ region of the *M. rossiaemeridionalis Xist*.
(DOC)

Table S2 Nucleotide sequences used for obtaining DNA probes for EMSA.
(DOC)

## References

[1] Heard E (2004). Recent advances in X-chromosome inactivation.. Curr Opin Cell Biol.

[2] Zakharova IS, Shevchenko AI, Zakian SM (2009). Monoallelic gene expression in mammals.. Chromosoma.

[3] Tattermusch A, Brockdorff N (2011). A scaffold for X chromosome inactivation.. Human Genetics.

[4] Xu N, Tsai CL, Lee JT (2006). Transient homologous chromosome pairing marks the onset of X inactivation.. Science.

[5] Augui S, Filion GJ, Huart S, Nora E, Guggiari M (2007). Sensing X chromosome pairs before X inactivation via a novel X-pairing region of the Xic.. Science.

[6] Starmer J, Magnuson T (2009). A new model for random X chromosome inactivation.. Development.

[7] Navarro P, Pichard S, Ciaudo C, Avner P, Rougeulle C (2005). Tsix transcription across the Xist gene alters chromatin conformation without affecting Xist transcription: implications for X-chromosome inactivation.. Genes Dev.

[8] Sado T, Hoki Y, Sasaki H (2005). Tsix silences Xist through modification of chromatin structure.. Dev Cell.

[9] Zhao J, Sun BK, Erwin JA, Song JJ, Lee JT (2008). Polycomb proteins targeted by a short repeat RNA to the mouse X chromosome.. Science.

[10] Chow JC, Hall LL, Clemson CM, Lawrence JB, Brown CJ (2003). Characterization of expression at the human XIST locus in somatic, embryonal carcinoma, and transgenic cell lines.. Genomics.

[11] Migeon BR (2002). X chromosome inactivation: theme and variations.. Cytogenet Genome Res.

[12] Migeon BR, Chowdhury AK, Dunston JA, McIntosh I (2001). Identification of TSIX, encoding an RNA antisense to human XIST, reveals differences from its murine counterpart: implications for X inactivation.. Am J Hum Genet.

[13] Komura J, Sheardown SA, Brockdorff N, Singer-Sam J, Riggs AD (1997). In vivo ultraviolet and dimethyl sulfate footprinting of the 5′ region of the expressed and silent Xist alleles.. J Biol Chem.

[14] Pugacheva EM, Tiwari VK, Abdullaev Z, Vostrov AA, Flanagan PT (2005). Familial cases of point mutations in the XIST promoter reveal a correlation between CTCF binding and pre-emptive choices of X chromosome inactivation.. Hum Mol Genet.

[15] Sheardown SA, Newall AE, Norris DP, Rastan S, Brockdorff N (1997). Regulatory elements in the minimal promoter region of the mouse Xist gene.. Gene.

[16] Pillet N, Bonny C, Schorderet DF (1995). Characterization of the promoter region of the mouse Xist gene.. Proc Natl Acad Sci U S A.

[17] Hendrich BD, Plenge RM, Willard HF (1997). Identification and characterization of the human XIST gene promoter: implications for models of X chromosome inactivation.. Nucleic Acids Res.

[18] Zakian SM, Kulbakina NA, Meyer MN, Semenova LA, Bochkarev MN (1987). Non-random inactivation of the X-chromosome in interspecific hybrid voles.. Genet Res.

[19] Nesterova TB, Johnston CM, Appanah R, Newall AE, Godwin J (2003). Skewing X chromosome choice by modulating sense transcription across the Xist locus.. Genes Dev.

[20] Newall AE, Duthie S, Formstone E, Nesterova T, Alexiou M (2001). Primary non-random X inactivation associated with disruption of Xist promoter regulation.. Hum Mol Genet.

[21] Nesterova TB, Slobodyanyuk SY, Elisaphenko EA, Shevchenko AI, Johnston C (2001). Characterization of the genomic Xist locus in rodents reveals conservation of overall gene structure and tandem repeats but rapid evolution of unique sequence.. Genome Res.

[22] Shevchenko AI, Malakhova AA, Elisaphenko EA, Mazurok NA, Nesterova TB (2011). Variability of sequence surrounding the Xist gene in rodents suggests taxon-specific regulation of X chromosome inactivation.. PLoS ONE.

[23] Ryan WA, Franza BR, Gilman MZ (1989). Two distinct cellular phosphoproteins bind to the c-fos serum response element.. EMBO J.

[24] Jones KA, Kadonaga JT, Rosenfeld PJ, Kelly TJ, Tjian R (1987). A cellular DNA-binding protein that activates eukaryotic transcription and DNA replication.. Cell.

[25] Turker MS (2002). Gene silencing in mammalian cells and the spread of DNA methylation.. Oncogene.

[26] Dementyeva EV, Shevchenko AI, Anopriyenko OV, Mazurok NA, Elisaphenko EA (2010). Difference between random and imprinted X inactivation in common voles.. Chromosoma.

[27] Renda M, Baglivo I, Burgess-Beusse B, Esposito S, Fattorusso R (2007). Critical DNA binding interactions of the insulator protein CTCF: a small number of zinc fingers mediate strong binding, and a single finger-DNA interaction controls binding at imprinted loci.. J Biol Chem.

[28] Kanduri C, Pant V, Loukinov D, Pugacheva E, Qi CF (2000). Functional association of CTCF with the insulator upstream of the H19 gene is parent of origin-specific and methylation-sensitive.. Curr Biol.

[29] Lee TI, Johnstone SE, Young RA (2006). Chromatin immunoprecipitation and microarray-based analysis of protein location.. Nat Protoc.

[30] Toth J, Biggin MD (2000). The specificity of protein-DNA crosslinking by formaldehyde: in vitro and in drosophila embryos.. Nucleic Acids Res.

[31] Orlando V (2000). Mapping chromosomal proteins in vivo by formaldehyde-crosslinked-chromatin immunoprecipitation.. Trends Biochem Sci.

[32] Kaczynski J, Zhang JS, Ellenrieder V, Conley A, Duenes T (2001). The Sp1-like protein BTEB3 inhibits transcription via the basic transcription element box by interacting with mSin3A and HDAC-1 co-repressors and competing with Sp1.. J Biol Chem.

[33] Zhou T, Chiang CM (2002). Sp1 and AP2 regulate but do not constitute TATA-less human TAF(II)55 core promoter activity.. Nucleic Acids Res.

[34] Peng Y, Jahroudi N (2003). The NFY transcription factor inhibits von Willebrand factor promoter activation in non-endothelial cells through recruitment of histone deacetylases.. J Biol Chem.

[35] Li G, Margueron R, Ku M, Chambon P, Bernstein BE (2010). Jarid2 and PRC2, partners in regulating gene expression.. Genes Dev.

[36] Landeira D, Sauer S, Poot R, Dvorkina M, Mazzarella L (2010). Jarid2 is a PRC2 component in embryonic stem cells required for multi-lineage differentiation and recruitment of PRC1 and RNA Polymerase II to developmental regulators.. Nat Cell Biol.

[37] Malin S, Linderson Y, Almqvist J, Ernberg I, Tallone T (2005). DNA-dependent conversion of Oct-1 and Oct-2 into transcriptional repressors by Groucho/TLE.. Nucleic Acids Res.

[38] Nagpal S, Ghosn C, DiSepio D, Molina Y, Sutter M (1999). Retinoid-dependent recruitment of a histone H1 displacement activity by retinoic acid receptor.. J Biol Chem.

[39] Allenby G, Bocquel MT, Saunders M, Kazmer S, Speck J (1993). Retinoic acid receptors and retinoid X receptors: interactions with endogenous retinoic acids.. Proc Natl Acad Sci U S A.

[40] Yasui D, Miyano M, Cai S, Varga-Weisz P, Kohwi-Shigematsu T (2002). SATB1 targets chromatin remodelling to regulate genes over long distances.. Nature.

[41] Pavan Kumar P, Purbey PK, Sinha CK, Notani D, Limaye A (2006). Phosphorylation of SATB1, a global gene regulator, acts as a molecular switch regulating its transcriptional activity in vivo.. Mol Cell.

[42] Chow J, Heard E (2009). X inactivation and the complexities of silencing a sex chromosome.. Curr Opin Cell Biol.

[43] Savarese F, Davila A, Nechanitzky R, De La Rosa-Velazquez I, Pereira CF (2009). Satb1 and Satb2 regulate embryonic stem cell differentiation and Nanog expression.. Genes Dev.

[44] Agrelo R, Souabni A, Novatchkova M, Haslinger C, Leeb M (2009). SATB1 defines the developmental context for gene silencing by Xist in lymphoma and embryonic cells.. Dev Cell.

[45] Quinlan KG, Nardini M, Verger A, Francescato P, Yaswen P (2006). Specific recognition of ZNF217 and other zinc finger proteins at a surface groove of C-terminal binding proteins.. Mol Cell Biol.

[46] Sado T, Ferguson-Smith AC (2005). Imprinted X inactivation and reprogramming in the preimplantation mouse embryo.. Hum Mol Genet.

[47] Johnsen SA, Gungor C, Prenzel T, Riethdorf S, Riethdorf L (2009). Regulation of estrogen-dependent transcription by the LIM cofactors CLIM and RLIM in breast cancer.. Cancer Res.

[48] Jonkers I, Barakat TS, Achame EM, Monkhorst K, Kenter A (2009). RNF12 is an X-encoded dose-dependent activator of X chromosome inactivation.. Cell.

[49] Barakat TS, Gunhanlar N, Gontan Pardo C, Achame EM, Ghazvini M (2011). RNF12 Activates Xist and Is Essential for X Chromosome Inactivation.. PLoS Genet.

[50] Cleynen I, Van de Ven WJ (2008). The HMGA proteins: a myriad of functions (Review).. Int J Oncol.

[51] Frazer KA, Pachter L, Poliakov A, Rubin EM, Dubchak I (2004). VISTA: computational tools for comparative genomics.. Nucleic Acids Res.

[52] Larkin MA, Blackshields G, Brown NP, Chenna R, McGettigan PA (2007). Clustal W and Clustal X version 2.0.. Bioinformatics.

[53] Cartharius K, Frech K, Grote K, Klocke B, Haltmeier M (2005). MatInspector and beyond: promoter analysis based on transcription factor binding sites.. Bioinformatics.

[54] Wingender E, Chen X, Fricke E, Geffers R, Hehl R (2001). The TRANSFAC system on gene expression regulation.. Nucleic Acids Res.

[55] Nesterova TB, Mazurok NA, Matveeva NM, Shilov AG, Yantsen EI (1994). Demonstration of the X-linkage and order to the genes GLA, G6PD, HPRT, and PGK in two vole species of the genus Microtus.. Cytogenet Cell Genet.

[56] Mazurok NA, Rubtsova NV, Grigor'eva EV, Matveeva NM, Zhelezova AI (2003). Isolation of ES-like lines of common voles of the genus Microtus from blastocysts and germ cells and as a result of the fusion of somatic cells with mouse embryonic stem cells.. Ontogenez.

[57] Sierra F, Tian J-M, Schibler U, Hames BD, Higgins SJ (1993). In vitro transcription with nuclear extracts from differentiated tissues.. Gene transcription: a practical approach.

[58] Dignam JD, Lebovitz RM, Roeder RG (1983). Accurate transcription initiation by RNA polymerase II in a soluble extract from isolated mammalian nuclei.. Nucleic Acids Research.

[59] Grunau C, Schattevoy R, Mache N, Rosenthal A (2000). MethTools–a toolbox to visualize and analyze DNA methylation data.. Nucleic Acids Res.

[60] Kim TH, Abdullaev ZK, Smith AD, Ching KA, Loukinov DI (2007). Analysis of the vertebrate insulator protein CTCF-binding sites in the human genome.. Cell.

