# Supplementary material for: A Regulatory Potential of the Xist Gene Promoter in Vole M. rossiaemeridionalis
Source: PLoS One. 2012 May 11;7(5):e33994. doi: 10.1371/journal.pone.0033994 (PMC3350511; doi:10.1371/journal.pone.0033994)

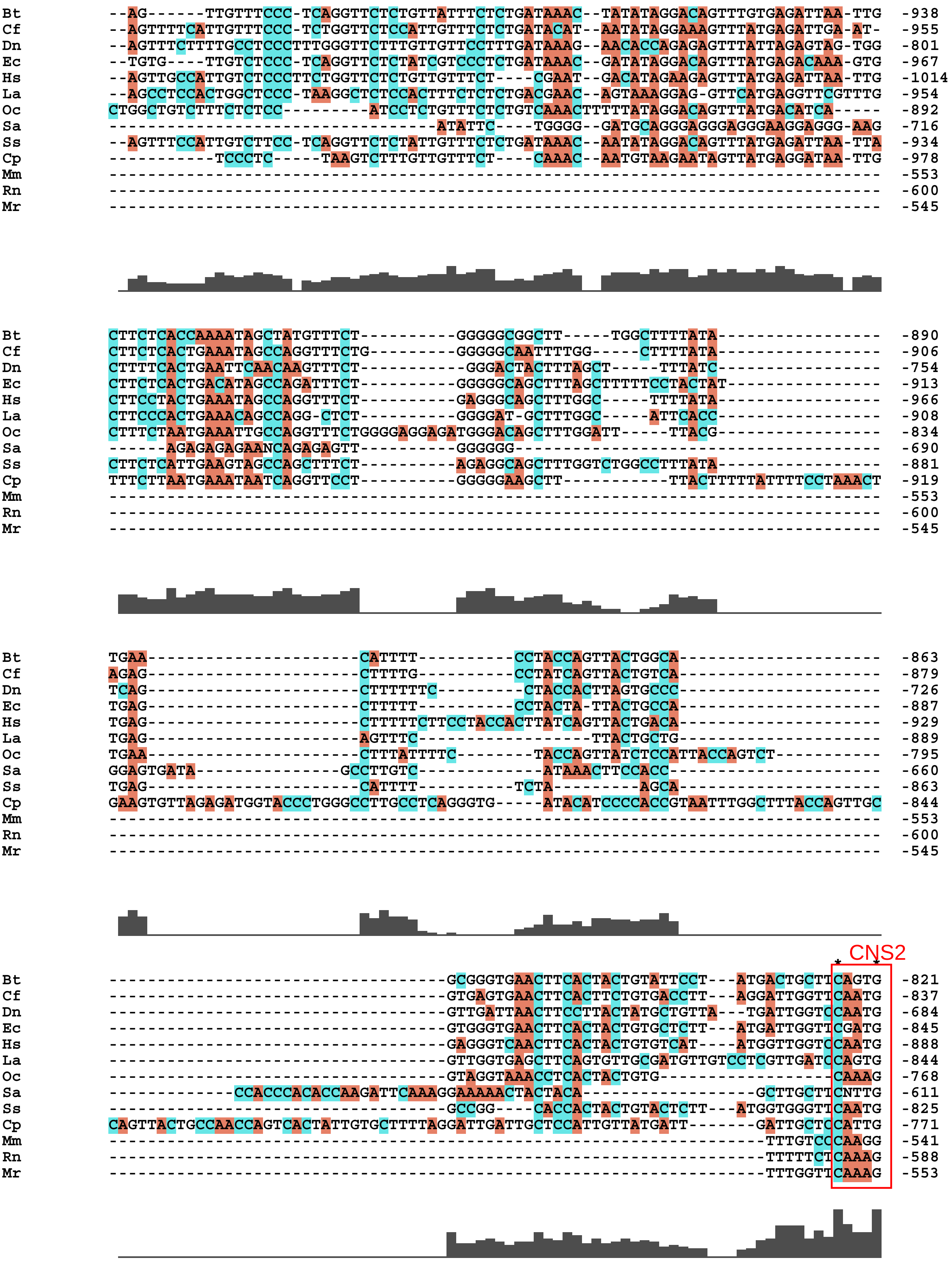
Bt --AG------TTGTTTCCC-TCAGGTTCTCTGTTATTTCTCTGATAAAC--TATATAGGACAGTTTGTGAGATTAA-TTG -938
Cf --AGTTTCATTGTTTCCC-TCTGGTTCTCCATTGTTTCTCTGATACAT--AATATAGGAAAGTTTATGAGATTGA-AT- -955
Dn --AGTTTCTTTTGCCTCCCTTTGGGTTCTTTGTTGTTCCTTTGATAAAG--AACACCAGAGAGTTTATTAGAGTAG-TGG -801
Ec --TGTG----TTGTCTCCC-TCAGGTTCTCTATCGTCCCTCTGATAAAC--GATATAGGACAGTTTATGAGACAAA-GTG -967
Hs --AGTTGCCATTGTCTCCCTTCTGGTTCTCTGTTGTTTCT----CGAAT--GACATAGAAGAGTTTATGAGATTAA-TTG -1014
La --AGCCTCCACTGGCTCCC-TAAGGCTCTCCACTTTCTCTCTGACGAAC--AGTAAAGGAG-GTTCATGAGGTTCGTTTG -954
Oc CTGGCTGTCTTTCTCTCC---------ATCCTCTGTTTCTCTGTCAAACTTTTTATAGGACAGTTTATGACATCA----- -892
Sa ------------------------------------ATATTC----TGGGG--GATGCAGGGAGGGAGGGAAGGAGGG-AAG -716
Ss --AGTTTCCATTGTCTTCC-TCAGGTTCTCTATTGTTTCTCTGATAAAC--AATATAGGACAGTTTATGAGATTAA-TTA -934
Cp -----------TCCCTC-----TAAGTCTTTGTTGTTTCT----CAAAC--AATGTAAGAATAGTTATGAGGATAA-TTG -978
Mm -- -553
Rn -- -600
Mr -- -545
Bt CTTCTCACCAAAATAGCTATGTTTCT----------GGGGGCGGCTT-----TGGCTTTTATA----------------- -890
Cf CTTCTCACTGAAATAGCCAGGTTTCTG---------GGGGGCAATTTTGG----CTTTTATA----------------- -906
Dn CTTTTCACTGAATTCAACAAGTTTCT-----------GGGACTACTTTAGCT-----TTTATC----------------- -754
Ec CTTCTCACTGACATAGCCAGATTTCT----------GGGGGCAGCTTTAGCTTTTTCCTACTAT---------------- -913
Hs CTTCCTACTGAAATAGCCAGGTTTCT----------GAGGGCAGCTTTGGC-----TTTTATA----------------- -966
La CTTCCCACTGAAACAGCCAGG-CTCT----------GGGGAT-GCTTTGGC-----ATTCACC----------------- -908
Oc CTTTCTAATGAAATTGCCAGGTTTCTGGGGAGGAGATGGGACAGCTTTGGATT-----TTACG----------------- -834
Sa ------AGAGAGAGAANCAGAGAGTT----------GGGGGG-------------------------------------- -690
Ss CTTCTCATTGAAGTAGCCAGCTTTCT----------AGAGGCAGCTTTGGTCTGGCCTTTATA----------------- -881
Cp TTTCTTAATGAAATAATCAGGTTCCT----------GGGGGAAGCTT-----------TTACTTTTTATTTTCCTAAACT -919
Mm -- -553
Rn -- -600
Mr -- -545
Bt TGAA----------------------CATTTT----------CCTACCAGTTACTGGCA--------------------- -863
Cf AGAG----------------------CTTTTG----------CCTATCAGTTACTGTCA--------------------- -879
Dn TCAG----------------------CTTTTTTC---------CTACCACTTAGTGCCC--------------------- -726
Ec TGAG----------------------CTTTTT----------CCTACTA-TTACTGCCA--------------------- -887
Hs TGAG----------------------CTTTTTCTTCCTACCACTTATCAGTTACTGACA--------------------- -929
La TGAG----------------------AGTTTC------------------TTACTGCTG--------------------- -889
Oc TGAA----------------------CTTTATTTTC--------TACCAGTTATCTCCATTACCAGTCT----------- -795
Sa GGAGTGATA-----------------GCCTTGTC-------------ATAAACTTCCACC-------------------- -660
Ss TGAG----------------------CATTTT----------TCTA--------AGCA---------------------- -863
Cp GAAGTGTTAGAGATGGTACCCTGGGCCTTGCCTCAGGGTG-----ATACATCCCCACCGTAATTTGGCTTTACCAGTTGC -844
Mm -- -553
Rn -- -600
Mr -- -545
CNS2
Bt -----------------------------------GCGGGTGAACTTCACTACTGTATTCCT---ATGACTGCTTCAGTG -821
Cf -----------------------------------GTGAGTGAACTTCACTTCTGTGACCTT---AGGATTGGTTCAATG -837
Dn -----------------------------------GTTGATTAACTTCCTTACTATGCTGTTA---TGATTGGTCCAATG -684
Ec -----------------------------------GTGGGTGAACTTCACTACTGTGCTCTT---ATGATTGGTTCGATG -845
Hs -----------------------------------GAGGGTCAACTTCACTACTGTGTCAT----ATGGTTGGTCCAATG -888
La -----------------------------------GTTGGTGAGCTTCAGTGTTGCGATGTTGTCCTCGTTGATCCAGTG -844
Oc -----------------------------------GTAGGTAAACCTCACTACTGTG------------------CAAAG -768
Sa -------------CCACCCACACCAAGATTCAAAGGAAAAACTACTACA-----------------GCTTGCTTCNTTG -611
Ss -----------------------------------GCCGG----CACCACTACTGTACTCTT---ATGGTGGGTTCAATG -825
Cp CAGTTACTGCCAACCAGTCACTATTGTGCTTTTAGGATTGATTGCTCCATTGTTATGATT-------GATTGCTCCATTG -771
Mm --TTTGTCCCAAGG -541
Rn --TTTTTCTCAAAG -588
Mr --TTTGGTTCAAAG -553

CNS2

```
                   *      *
Bt  TT-GGGGTTGTCAGATTCACGGAGTAAGTAGAAAAAA----TACT---------------GAAGATTGAG----------  -771
Cf  TTGGGGGTCATCAGATTCATGGAGGAAATAG-GAAAA----TACTA--------------AAAGATTAAG----------  -786
Dn  TT-GGAGTCTTCAGATTCTTAGAGAATGT-----AGGCAACTACT---------------AAAGACTGAG----------  -635
Ec  TT-GGGGTCATCGGATTCACGGAGAAAGTAGA-AAAA----TACT---------------AAAGATTAAG----------  -796
Hs  TT-GGGTTCCTCAGATTCATGGAGGAAATA-GGAAAA----TAGT---------------AAAGATTAAG----------  -839
La  TT-GAGTAGAGCAAATC----GACGAAGTA-AGCAAA----TACT---------------AAAGATGAAG----------  -799
Oc  TT-GGAGCTATCAGATTCAACGAAGTAG--------------------------------AAAAGTAAGG----------  -731
Sa  GT-GTTGTT------TTCAAGTACA-----------------------------------------CGCT----------  -589
Ss  TT-GGGGTCATCAGATTCATGGAAGAAG--GAGAAGA----TACT---------------AAAGATGAAG----------  -777
Cp  TT-ATGATTGATTG-TTCATGGAGG--------------------AAGAAATAAT-----AAAGATCAAG----------  -728
Mm  TATGGAGTCACCAGGTTCCCAGA------------------------------------GAAAGACTAAGATG-------  -504
Rn  TTTGGAGCCATCAGTTTCCTGGAA----------------------------ACTGGATGAAAGACTAAAATGACCAAGC  -536
Mr  TTTGGAGTTACCAGATTCCCGGA-------------------------------------AAGTACAAAAATGA------  -496
```

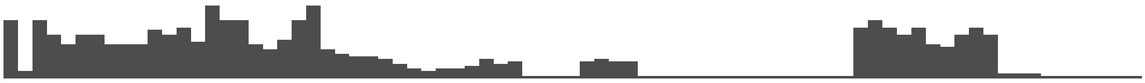

```
Bt  -----------------------------------------------GGTTCACAGTTTAAAAGTTACAAGTAATATA--  -740
Cf  -----------------------------------------------GACTCACGGTTTAAAAATTGCAAACAGATCACA  -753
Dn  -----------------------------------------------AATTCACAGTTTAAACATTACAAAGAGGTT---  -605
Ec  -----------------------------------------------GATTCGCAGTTTAAAACTTACAAACAGGTCAAA  -763
Hs  -----------------------------------------------GAGTCATAGTTAAAAAATTACAAACAGGTCACA  -806
La  -----------------------------------------------GAAATACAGTTTCAGAGTTAAAAACTGGT----  -770
Oc  -----------------------------------------------AAGTAAAAGTTAGGGACTCACTAACTGCTGACA  -698
Sa  -----------------------------------------------GACCAGTAA-TTTAAAATT-----CCTCTTACA  -562
Ss  -----------------------------------------------GTTTCACAGTTTAAAA--------CATTACA--  -754
Cp  -----------------------------------------------GACTAATAGCTTTAAAAATAGAAACAGGTCACA  -695
Mm  -----------------------------------CCAATGACCCAAAACTATGAGCGTAAGCCCTCCAAATCGGTCACA  -459
Rn  GCAAAATTCCAAGCGTAAAATTCAAGACGTAAAAATCCAAGACGCAAAATTCCAAGCGTAAGTCCCGCAAATAGGTCACA  -456
Mr  ------------------------------------TACAGAACCAAAATTCTGTGCGCAATCAGTACAAATAGGTCACA  -452
```

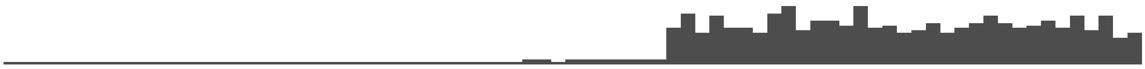

```
Bt  ----------------GATTGGTTAGG-GGCTAAATCAGCATTCTGTGAAATGATT---TTTCTTTCCT---CTCTCTT-  -684
Cf  GACAGTACAGCTTCTTGATTATTTAGT-GGCCAAAATGACATTCTGTGAATTACCTTCTGTTCCTTCCT---TTCTCTT-  -678
Dn  -ACAGTTTTCCTCCTGGATTACTTAAGTGGTCAAATGGCCATTCTTTGATCTGTCTT----CCTTTACG---TCCTCTT-  -534
Ec  AACAGTACTC------GATTATTTCGG-GGCCAAATTGGCATTCTGTGAAATGCCTTCCTTTCCTTCCT---CTCTTTT-  -694
Hs  AACAGTACTCTTTCTTGATTATTTAGG-AACCAAATAGCCATTCTATGAAATGTCTTTCTTTCCTTTTT---CTCTCTT-  -731
La  GGCAGTGTTGCTTATCAATTGTTTAGG-GGCCAAATAGCCTTTCTGTGAGATG-----CGTTCCTGTGT---CTCTCCG-  -700
Oc  GAC--------------------TAGG-GACCACACAGCCATTCTGTTAAATGTC-----TACCTGACTGTCTTCTTTT-  -645
Sa  A-----ATTTCT-------------------CAATTG-------------------------------------TTCCT-  -544
Ss  ----------------GTTTACTCAGG-GGCTACATGGACTTCCTGTGAAATGTCT---TTTCCTCCCT---CTGTCTT-  -698
Cp  AACAGCACTTCT-------------------CGATTAGAGAC-------------------------------CTCCTT-  -666
Mm  ACT-----AATACTCTGATAATTTAGG-AACCAAGGAGCCATTTTGTGAGGCATTTCTACCCTTTCCTCT-----CCTCA  -390
Rn  ACC-----AGTACTCTGATAATTTAGG-AATCACGGAGCCATTTTGTGAGACATTCCCACCCTTTCCTGT-----CCTGG  -387
Mr  GCCAA--TAATACCCTAATAATTTAGG-AACCAAGGAACGATTTTGTGAAGCACCTCTTCTTTTTCCTTA-----CCTC-  -381
```

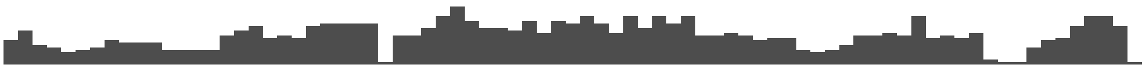

```
Bt  -----GCTC--------ACT------TAGAAACA-------CT----TCCATTCTTCTATTTTCAGGGGCCAC---AGAA  -637
Cf  -----GCTCACCGTTTGACT------TAAAAGCA-------CT----TCTACATCTCCATGATCGGGGGCCAT---ACAA  -623
Dn  -----GCTCACCAATTGACT------C--AAGTA-------TT----TTCTTTCTTCTATGGTCAGGGGTCAT---AC-A  -482
Ec  -----GCTTATCAATTAACT------TATAAGCA-------CT----TCTAATCGCCTGCGATCAGGGGCCAT---ACAA  -639
Hs  -----GCTCACCAATTGACT------CGTAAGCA-------CT----TCCGTTCTCTTATGGTTGGGAGCCAT---AC-A  -677
La  -----AC-----------CG------CATAAGTG-------CTTAACTCACCTTTCCAATTGTCGGGCGCCAC---AG-C  -653
Oc  -----GTTAATCAATTGGCTG-----CAAAAGCA-------GTTAG----AGTCTCTTATCACCTGGGGCTATGG-G---  -590
Sa  -----NGCCACACCTGGTAT------TACTCAGGAGTTGATCT---------------------------------A---  -511
Ss  -----GCTCACCAGTTAACT------TAGAAGCG-------TT----TCCAGTCTCCTATGATCAGCAGCCAT---GAAA  -643
Cp  -----AGTTACCAATTGAATCCTAAATACTTATGGTCTGGTCTC-------------CATGAATAGGGGCCACCCAA---  -607
Mm  TCTGAGGTCGCCAACTAATG------CAGAAGAAC------------TTCTAGTGTCCAAGAC-CGGAGCGA----CACA  -333
Rn  ATTGAGCTTACCACCTAATT------CATGGGAACTTT---------TTCTTGTCTCCAAGACGCGGAGCGA----TACA  -326
Mr  ---GAGCCCACCAATTGATC------CGTAAAGCA------------TTTTAGTCACCAAGACGAGGGGCTA----TGCA  -326
```

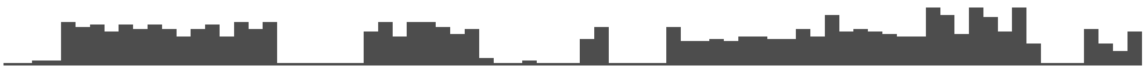

```
        *
Bt  ATGC--AGTGCTGGAGTAGAAAATACACTGA-----CTCAGA---------A--------TTAC--AGTATGTGACT---  -586
Cf  AGGCAGAATGCTGGAGTAGAAAATACACGGA-----CTCAAA---------A--------TCAT--GGTGTGTGACC---  -570
Dn  AGGCAGAGTGATGGAGTAGAAAATAAATTCA-----ATCTGA---------A--------TCATG--GTGTGTGACT---  -429
Ec  AGACTTCGTGTTGGAGTAGAAAATTTACTGT-----CTCAGA---------A--------TCAT--AGCGTGCGACC---  -586
Hs  AGGTAGAGTGTTGGAGTAAAAACTACATTGA-----CTCTGA---------A--------TCATG--GTGTGTGACC---  -624
La  GGGCCCTGTACTGGGGTACAAGGAACACTCA-----ATCGCA---------G--------CC----------TGAGT---  -608
Oc  AGACAAAGTATTGGAGTAGAAAATGCAGGGA-----CTGGGG---------AGTCGAGGGT--------GTGTGACT---  -535
Sa  -GGCTAAGTGT-----------ATATATCTT-----TTTTATGTTTTGGTCC----------------------ACCTGG  -470
Ss  AGGCAGAGTGATGCAGTAGAAAATACACTGA-----CTCAGA---------A--------TCTCTTGGTGTGTGACT---  -588
Cp  -GGTAAGGTGTTGAAAAAGTAAATACACTAA-----CTTGGAGTCATGGTATGT-------------------GACCTAG  -552
Mm  TGGTT---TGTCCAAGTAGAAGATATATTGAAATTTTGCATAGA------------------------------------  -292
Rn  AGGCCGGATGTCCAAGTAGAAGATATGTTGAAAATTTGCATAGA------------------------------------  -282
Mr  AGGTTGGATGTCCAAGTAGACGATATATTGAAAATATGCATA--------------------------------------  -284
```

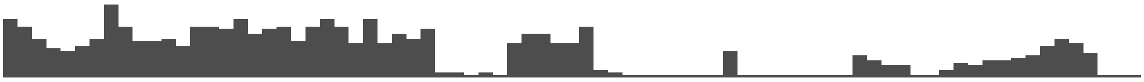

```
Bt  ------TTG---------------AGCACATCATGTAAC------------CTTTATGGGTCTC----------TTTTCC  -549
Cf  ------TCA---------------TGCATATCATATAACAG----------CTTCAT---TATC----------TATCAA  -534
Dn  ------TCATC-------------CACCCATT-------------------TTTTATGGGCTTCTATCTTGTAATATATA  -387
Ec  ------TCA---------------TGTACGTCGTGTAAC------------CTTTCTGACAACC----------TATAAA  -549
Hs  ------TCG---------------GGCACATGATGTAAC------------CTCTATAGGACTCTATTTTAA--TGTATA  -579
La  ------CCA---------------TGTACATTACTCAGC------------CTTTACGGCCCTC----------TGTATA  -571
Oc  ------TCA---------------TGCACATAATTTCACCTTTGTTTCCTAATCTATGA---------------------  -497
Sa  CAAAGCTCAGCG------------TTTACTCCTTG----------------CTCTACA----------------CTCA--  -436
Ss  ------TTGT--------------TGCACATCATGTAAC------------CTTTATGGGTCTC----------TTTTTC  -550
Cp  TGAACTTCA---------------TTTAACCTTTATGGAT-----------TTCTGT-----------------CTCATA  -515
Mm  -------CA-GGTGTGTG-CTAAATGTACACACCCAAATGTTTA----------TGTGAAGTTC----------TACATA  -241
Rn  -------CATGGTGTGTGACCTAGTGTACATTATTTAACGTTTA----------TGGATAG-------------TGTGCA  -232
Mr  --------------------------TACATTATACAACGTTTA----------TGTGCCTTTCC---------TATA--  -251
```

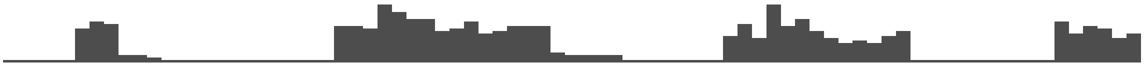

```
Bt  ------G--ACTAAACCTTCA------------------------------CTATCACATTAGATAACGGAAGGGAAAGT  -507
Cf  ACATAGG--AACAAACGGGCA------------------------------CTATCTCATTAGATAATGAAAGGGAAAGT  -486
Dn  AAACAGAGGGATAACCCTTTA------------------------------CTATCACA--GGAGAATGGAAGGGAAA--  -341
Ec  ACAAAGG--ACTAGACCTTCA------------------------------CTATCGCCTTAGGTTATGGAAGGGGAAGT  -501
Hs  AAACAGG--AATAATCCTTTATTATTATCTATGCAATACATATTCCATTATCTATTACATGGGATAATGGAATGGGAAGT  -501
La  AGGCAGGTAAATCATTCTTCA------------------------------CTACCGCATTCGATGATGCTA-GCGAAGT  -522
Oc  ----------ATGATTCTTCAC--------------------------------CTGTATTTGGTAATGGAAGAGAAAGT  -459
Sa  --GTA------TTACCTTCTGG----------------------------------------------------------  -422
Ss  ------A--AGTAAACCTTCA------------------------------CTATCACGTTAGATAATGGAAGGGAAAGT  -508
Cp  ATGTA------TAATCCTCTAGT-----------------------------------ATCAGATAACGGAAAGGAAACT  -476
Mm  AAC-----------------------------------------------------------------------------  -238
Rn  AA------------------------------------------------------------------------------  -230
Mr  --------------------------------------------------------------------------------  -251
```

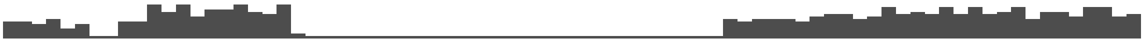

```
Bt  CCCCTG-AAGAGGGTTCTAACCTTAATGTATTGCCCCTTCCTAGCTTCTTTGGTTCCTATGTCTGGTGG----ACAAGTT  -432
Cf  CCTTTG-GAGAGGGTTCTAACCTTAATGTAGTAC-CCTTCCTAGCTG----------------TGGTGG----GGGAGTT  -428
Dn  --------------------------------------TTTTAGCTAATTTGATCCAG----------------------  -321
Ec  CCCTTG-AAGAGGGTTCTAACCTTAATGTATTGCTCCTTCCTAGCTTCTTTGGTTCAAAAGTTTGGTAG----GGAAGTT  -426
Hs  CCCTTG-AAGATGGTACTAACCTCAATGTATTACTCCTTTCTAGCTTCTTTGGTTCAAAAGTTTGGTGG----AGGAGTT  -426
La  GCTTTGAAAGGTGGTTC-AACCTCGATGTACTGCCGCTTCCTCACTTCTTTGATTCAAAGGTTTGGTCA----GGAGGTT  -447
Oc  CCTTGG-AAGATGGCTCTAACCTTAAGGTACTACCCTTTCTTAACTTCCTTGGTTCGAAAGTTTGGTGGTGGTGGGGATT  -380
Sa  ----------------------------------TGGTCCTCAG------GGGACCATATGC---ATGCT----AGGGAT  -389
Ss  CCTTTG-AAGAGGTTTCTAACCTTAATGTATTGCCCCTTCCTGGCTTCTTTGGTTCAAAAGTTTGGTGG----GGAAGTT  -433
Cp  CCTTTGAGA-ATGGTCTTAATCTTAATGTGTTATTGTTTCTTAGCTTCTTGGGTCCAAAAGTTTGGTGGT----AGAGCT  -401
Mm  ------------GTTTTAGCTGTAAAAT-----------------------------------------------AGGAT  -216
Rn  ------------------AGCTGTAAAAT----------------------------------------------AGGAT  -214
Mr  ------------------AGCTATAAAATAG--------------------------------------------AGGAT  -233
```

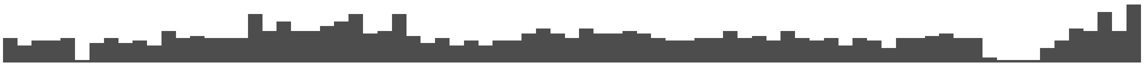

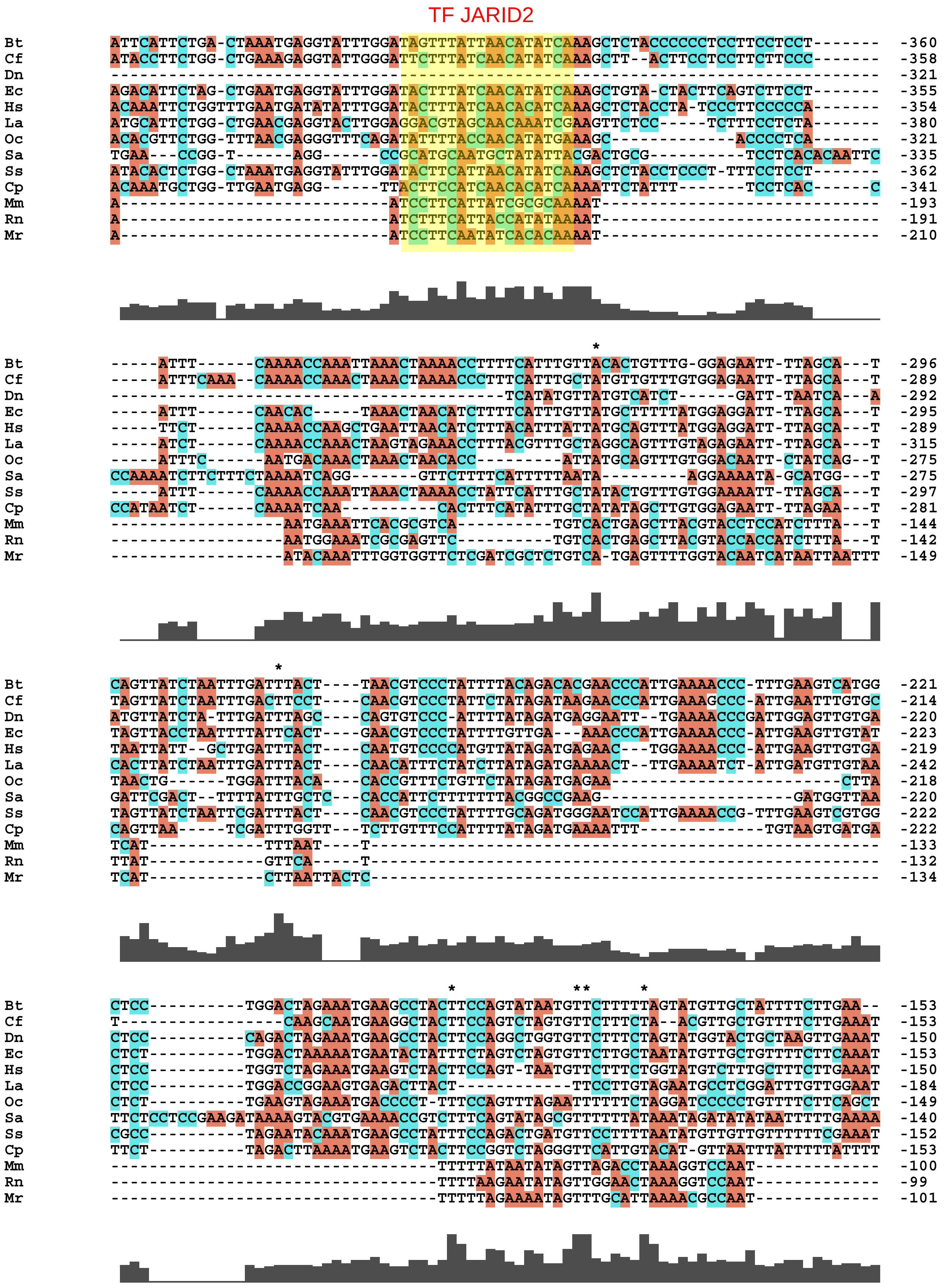
TF JARID2
Bt ATTCATTCTGA-CTAAATGAGGTATTTGGATAGTTTATTAACATATCAAAGCTCTACCCCCCTCCTTCCTCCT------- -360
Cf ATACCTTCTGG-CTGAAAGAGGTATTGGGATTCTTTATCAACATATCAAAGCTT--ACTTCCTCCTTCTTCCC------- -358
Dn -- -321
Ec AGACATTCTAG-CTGAATGAGGTATTTGGATACTTTATCAACATATCAAAGCTGTA-CTACTTCAGTCTTCCT------- -355
Hs ACAAATTCTGGTTTGAATGATATATTTGGATACTTTATCAACACATCAAAGCTCTACCTA-TCCCTTCCCCCA------- -354
La ATGCATTCTGG-CTGAACGAGGTACTTGGAGGACGTAGCAACAAATCGAAGTTCTCC-----TCTTTCCTCTA------- -380
Oc ACACGTTCTGG-TTTAACGAGGGTTTCAGATATTTTACCAACATATGAAAGC------------ACCCCTCA------- -321
Sa TGAA---CCGG-T------AGG------CCGCATGCAATGCTATATTACGACTGCG----------TCCTCACACAATTC -335
Ss ATACACTCTGG-CTAAATGAGGTATTTGGATACTTCATTAACATATCAAAGCTCTACCTCCCT-TTTCCTCCT------- -362
Cp ACAAATGCTGG-TTGAATGAGG------TTACTTCCATCAACACATCAAAATTCTATTT-------TCCTCAC------C -341
Mm A----------------------------ATCCTTCATTATCGCGCAAAT------------------------------ -193
Rn A----------------------------ATCTTTCATTACCATATAAAT------------------------------ -191
Mr A----------------------------ATCCTTCAATATCACACAAAT------------------------------ -210
*
Bt -----ATTT------CAAAACCAAATTAAACTAAAACCTTTTCATTTGTTACACTGTTTG-GGAGAATT-TTAGCA---T -296
Cf -----ATTTCAAA--CAAAACCAAACTAAACTAAAACCCTTTCATTTGCTATGTTGTTTGTGGAGAATT-TTAGCA---T -289
Dn --TCATATGTTATGTCATCT------GATT-TAATCA---A -292
Ec -----ATTT------CAACAC-----TAAACTAACATCTTTTCATTTGTTATGCTTTTTATGGAGGATT-TTAGCA---T -295
Hs -----TTCT------CAAAACCAAGCTGAATTAACATCTTTACATTTATTATGCAGTTTATGGAGGATT-TTAGCA---T -289
La -----ATCT------CAAAACCAAACTAAGTAGAAACCTTTACGTTTGCTAGGCAGTTTGTAGAGAATT-TTAGCA---T -315
Oc -----ATTTC------AATGACAAACTAAACTAACACC---------ATTATGCAGTTTGTGGACAATT-CTATCAG--T -275
Sa CCAAAATCTTCTTTCTAAAATCAGG-------GTTCTTTTCATTTTTAATA---------AGGAAAATA-GCATGG---T -275
Ss -----ATTT------CAAAACCAAATTAAACTAAAACCTATTCATTTGCTATACTGTTTGTGGAAAATT-TTAGCA---T -297
Cp CCATAATCT------CAAAATCAA----------CACTTTCATATTTGCTATATAGCTTGTGGAGAATT-TTAGAA---T -281
Mm -----------------AATGAAATTCACGCGTCA----------TGTCACTGAGCTTACGTACCTCCATCTTTA---T -144
Rn -----------------AATGGAAATCGCGAGTTC----------TGTCACTGAGCTTACGTACCACCATCTTTA---T -142
Mr -----------------ATACAAATTTGGTGGTTCTCGATCGCTCTGTCA-TGAGTTTGGTACAATCATAATTAATTT -149
*
Bt CAGTTATCTAATTTGATTTACT----TAACGTCCCTATTTTACAGACACGAACCCATTGAAAACCC-TTTGAAGTCATGG -221
Cf TAGTTATCTAATTTGACTTCCT----CAACGTCCCTATTCTATAGATAAGAACCCATTGAAAGCCC-ATTGAATTTGTGC -214
Dn ATGTTATCTA-TTTGATTTAGC----CAGTGTCCC-ATTTTATAGATGAGGAATT--TGAAAACCCGATTGGAGTTGTGA -220
Ec TAGTTACCTAATTTTATTCACT----GAACGTCCCTATTTTGTTGA---AAACCCATTGAAAACCC-ATTGAAGTTGTAT -223
Hs TAATTATT--GCTTGATTTACT----CAATGTCCCCATGTTATAGATGAGAAC---TGGAAAACCC-ATTGAAGTTGTGA -219
La CACTTATCTAATTTGATTTACT----CAACATTTCTATCTTATAGATGAAAACT--TTGAAAATCT-ATTGATGTTGTAA -242
Oc TAACTG------TGGATTTACA----CACCGTTTCTGTTCTATAGATGAGAA----------------------CTTA -218
Sa GATTCGACT--TTTTATTTGCTC---CACCATTCTTTTTTTACGGCCGAAG------------------GATGGTTAA -220
Ss TAGTTATCTAATTCGATTTACT----CAACGTCCCTATTTTGCAGATGGGAATCCATTGAAAACCG-TTTGAAGTCGTGG -222
Cp CAGTTAA-----TCGATTTGGTT---TCTTGTTTCCATTTTATAGATGAAAATTT-------------TGTAAGTGATGA -222
Mm TCAT-----------TTTAAT----T-- -133
Rn TTAT-----------GTTCA-----T-- -132
Mr TCAT-----------CTTAATTACTC-- -134
* ** *
Bt CTCC----------TGGACTAGAAATGAAGCCTACTTCCAGTATAATGTTCTTTTTAGTATGTTGCTATTTTCTTGAA-- -153
Cf T----------------CAAGCAATGAAGGCTACTTCCAGTCTAGTGTTCTTTCTA--ACGTTGCTGTTTTCTTGAAAT -153
Dn CTCC---------CAGACTAGAAATGAAGCCTACTTCCAGGCTGGTGTTCTTTCTAGTATGGTACTGCTAAGTTGAAAT -150
Ec CTCT----------TGGACTAAAAATGAATACTATTTCTAGTCTAGTGTTCTTGCTAATATGTTGCTGTTTTCTTCAAAT -153
Hs CTCC----------TGGTCTAGAAATGAAGTCTACTTCCAGT-TAATGTTCTTTCTGGTATGTCTTTGCTTTCTTGAAAT -150
La CTCC----------TGGACCGGAAGTGAGACTTACT------------TTCCTTGTAGAATGCCTCGGATTTGTTGGAAT -184
Oc CTCT----------TGAAGTAGAAATGACCCCT-TTTCCAGTTTAGAATTTTTTCTAGGATCCCCCTGTTTTCTTCAGCT -149
Sa TTCTCCTCCGAAGATAAAAGTACGTGAAAACCGTCTTTCAGTATAGCGTTTTTTATAAATAGATATATAATTTTTGAAAA -140
Ss CGCC----------TAGAATACAAATGAAGCCTATTTCCAGACTGATGTTCCTTTTAATATGTTGTTGTTTTTCGAAAT -152
Cp TTCT----------TAGACTTAAAATGAAGTCTACTTCCGGTCTAGGGTTCATTGTACAT-GTTAATTTATTTTTATTTT -153
Mm ---------------------------------TTTTTATAATATAGTTAGACCTAAAGGTCCAAT------------- -100
Rn ---------------------------------TTTTAAGAATATAGTTGGAACTAAAGGTCCAAT------------- -99
Mr ---------------------------------TTTTTAGAAAATAGTTTGCATTAAAACGCCAAT------------- -101

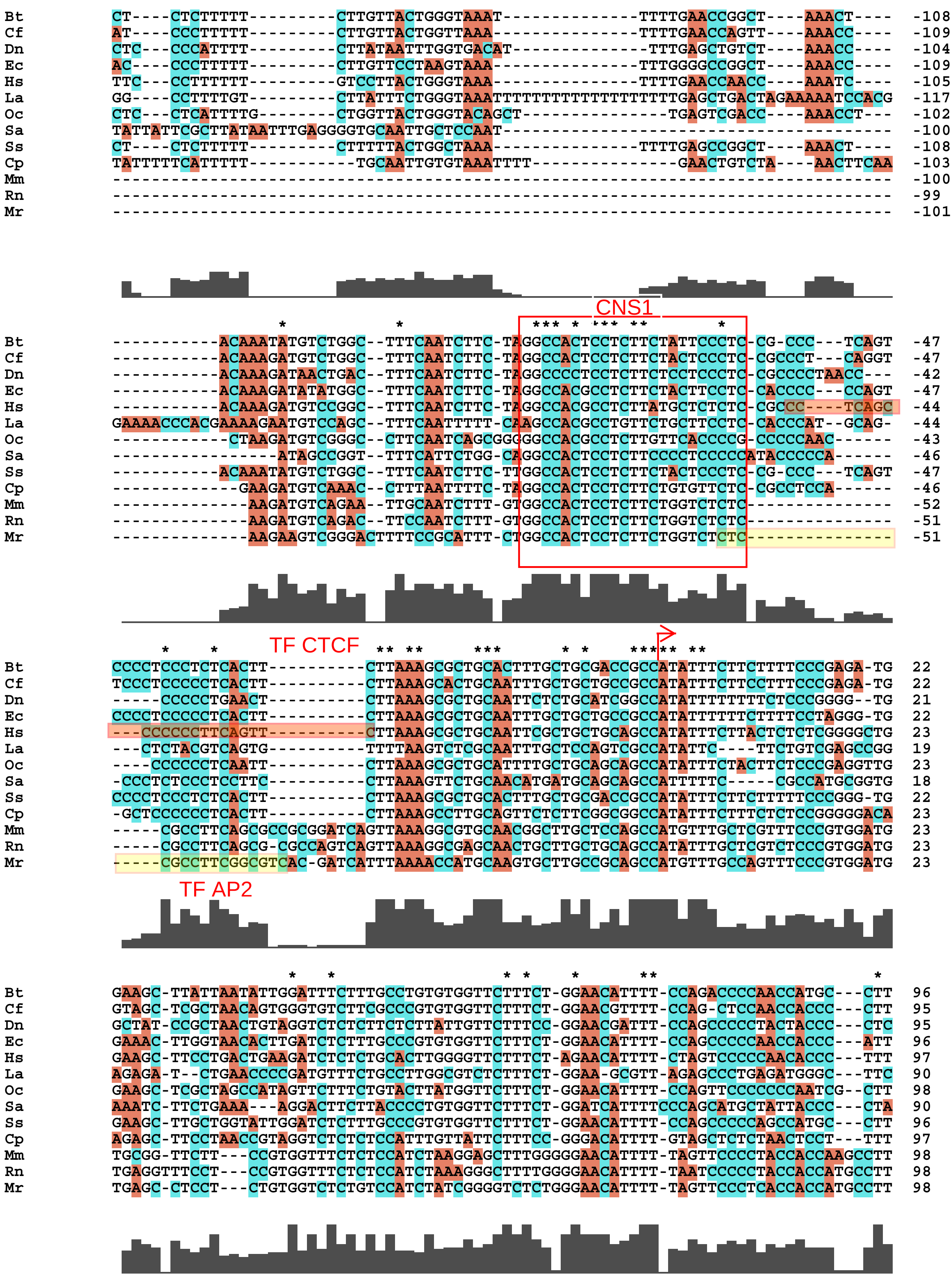
Bt CT----CTCTTTTT---------CTTGTTACTGGGTAAAT--------------TTTTGAACCGGCT----AAACT---- -108
Cf AT----CCCTTTTT---------CTTGTTACTGGTTAAA---------------TTTTGAACCAGTT----AAACC---- -109
Dn CTC---CCCATTTT---------CTTATAATTTGGTGACAT--------------TTTGAGCTGTCT----AAACC---- -104
Ec AC----CCCTTTTT---------CTTGTTCCTAAGTAAA---------------TTTGGGGCCGGCT----AAACC---- -109
Hs TTC---CCTTTTTT---------GTCCTTACTGGGTAAA---------------TTTTGAACCAACC----AAATC---- -105
La GG----CCTTTTGT---------CTTATTTCTGGGTAAATTTTTTTTTTTTTTTTTTGAGCTGACTAGAAAAATCCACG -117
Oc CTC---CTCATTTTG--------CTGGTTACTGGGTACAGCT---------------TGAGTCGACC----AAACCT--- -102
Sa TATTATTCGCTTATAATTTGAGGGGTGCAATTGCTCCAAT-- -100
Ss CT----CTCTTTTT---------CTTTTTACTGGCTAAA---------------TTTTGAGCCGGCT----AAACT---- -108
Cp TATTTTTCATTTTT-----------TGCAATTGTGTAAATTTT---------------GAACTGTCTA---AACTTCAA -103
Mm -- -100
Rn -- -99
Mr -- -101
CNS1
Bt -----------ACAAATATGTCTGGC--TTTCAATCTTC-TAGGCCACTCCTCTTCTATTCCCTC-CG-CCC---TCAGT -47
Cf -----------ACAAAGATGTCTGGC--TTTCAATCTTC-TAGGCCACTCCTCTTCTACTCCCTC-CGCCCT---CAGGT -47
Dn -----------ACAAAGATAACTGAC--TTTCAATCTTC-TAGGCCCCTCCTCTTCTCCTCCCTC-CGCCCCTAACC--- -42
Ec -----------ACAAAGATATATGGC--TTTCAATCTTC-TAGGCCACGCCTCTTCTACTTCCTC-CACCCC---CCAGT -47
Hs -----------ACAAAGATGTCCGGC--TTTCAATCTTC-TAGGCCACGCCTCTTATGCTCTCTC-CGCCC----TCAGC -44
La GAAAACCCACGAAAAGAATGTCCAGC--TTTCAATTTTT-CAAGCCACGCCTGTTCTGCTTCCTC-CACCCAT--GCAG- -44
Oc ------------CTAAGATGTCGGGC--CTTCAATCAGCGGGGGCCACGCCTCTTGTTCACCCCG-CCCCCAAC------ -43
Sa ----------------ATAGCCGGT--TTTCATTCTGG-CAGGCCACTCCTCTTCCCCTCCCCATACCCCCA------- -46
Ss -----------ACAAATATGTCTGGC--TTTCAATCTTC-TTGGCCACTCCTCTTCTACTCCCTC-CG-CCC---TCAGT -47
Cp -------------GAAGATGTCAAAC--CTTTAATTTTC-TAGCCACTCCTCTTCTGTGTTCTC-CGCCTCCA------ -46
Mm --------------AAGATGTCAGAA--TTGCAATCTTT-GTGGCCACTCCTCTTCTGGTCTCTC--------------- -52
Rn --------------AAGATGTCAGAC--TTCCAATCTTT-GTGGCCACTCCTCTTCTGGTCTCTC--------------- -51
Mr --------------AAGAAGTCGGGACTTTTCCGCATTT-CTGGCCACTCCTCTTCTGGTCTCTC--------------- -51
TF CTCF
Bt CCCCTCCCTCTCACTT----------CTTAAAGCGCTGCACTTTGCTGCGACCGCCATATTTCTTCTTTTCCCGAGA-TG 22
Cf TCCCTCCCCCTCACTT----------CTTAAAGCACTGCAATTTGCTGCTGCCGCCATATTTCTTCCTTTCCCGAGA-TG 22
Dn -----CCCCCTGAACT----------CTTAAAGCGCTGCAATTCTCTGCATCGGCCATATTTTTTTTCTCCCGGGG--TG 21
Ec CCCCTCCCCCTCACTT----------CTTAAAGCGCTGCAATTTGCTGCTGCCGCCATATTTTTTCTTTTCCTAGGG-TG 22
Hs ---CCCCCCTTCAGTT----------CTTAAAGCGCTGCAATTCGCTGCTGCAGCCATATTTCTTACTCTCTCGGGGCTG 23
La ---CTCTACGTCAGTG----------TTTTAAGTCTCGCAATTTGCTCCAGTCGCCATATTC----TTCTGTCGAGCCGG 19
Oc ----CCCCCCTCAATT----------CTTAAAGCGCTGCATTTTGCTGCAGCAGCCATATTTCTACTTCTCCCGAGGTTG 23
Sa -CCCTCTCCCTCCTTC----------CTTAAAGTTCTGCAACATGATGCAGCAGCCATTTTTC-----CGCCATGCGGTG 18
Ss CCCCTCCCTCTCACTT----------CTTAAAGCGCTGCACTTTGCTGCGACCGCCATATTTCTTCTTTTTCCCGGG-TG 22
Cp -GCTCCCCCCTCACTT----------CTTAAAGCCTTGCAGTTCTCTTCGGCGGCCATATTTCTTTCTCTCCGGGGGACA 23
Mm -----CGCCTTCAGCGCCGCGGATCAGTTAAAGGCGTGCAACGGCTTGCTCCAGCCATGTTTGCTCGTTTCCCGTGGATG 23
Rn -----CGCCTTCAGCG-CGCCAGTCAGTTAAAGGCGAGCAACTGCTTGCTGCAGCCATATTTGCTCGTCTCCCGTGGATG 23
Mr -----CGCCTTCGGCGTCAC-GATCATTTAAAACCATGCAAGTGCTTGCCGCAGCCATGTTTGCCAGTTTCCCGTGGATG 23
TF AP2
Bt GAAGC-TTATTAATATTGGATTTCTTTGCCTGTGTGGTTCTTTCT-GGAACATTTT-CCAGACCCCAACCATGC---CTT 96
Cf GTAGC-TCGCTAACAGTGGGTGTCTTCGCCCGTGTGGTTCTTTCT-GGAACGTTTT-CCAG-CTCCAACCACCC---CTT 95
Dn GCTAT-CCGCTAACTGTAGGTCTCTCTTCTCTTATTGTTCTTTCC-GGAACGATTT-CCAGCCCCCTACTACCC---CTC 95
Ec GAAAC-TTGGTAACACTTGATCTCTTTGCCCGTGTGGTTCTTTCT-GGAACATTTT-CCAGCCCCCAACCACCC---ATT 96
Hs GAAGC-TTCCTGACTGAAGATCTCTCTGCACTTGGGGTTCTTTCT-AGAACATTTT-CTAGTCCCCCAACACCC---TTT 97
La AGAGA-T--CTGAACCCCGATGTTTCTGCCTTGGCGTCTCTTTCT-GGAA-GCGTT-AGAGCCCTGAGATGGGC---TTC 90
Oc GAAGC-TCGCTAGCCATAGTTCTTTCTGTACTTATGGTTCTTTCT-GGAACATTTT-CCAGTTCCCCCCCAATCG--CTT 98
Sa AAATC-TTCTGAAA---AGGACTTCTTACCCCTGTGGTTCTTTCT-GGATCATTTTCCCAGCATGCTATTACCC---CTA 90
Ss GAAGC-TTGCTGGTATTGGATCTCTTTGCCCGTGTGGTTCTTTCT-GGAACATTTT-CCAGCCCCCAGCCATGC---CTT 96
Cp AGAGC-TTCCTAACCGTAGGTCTCTCTCCATTTGTTATTCTTTCC-GGGACATTTT-GTAGCTCTCTAACTCCT---TTT 97
Mm TGCGG-TTCTT---CCGTGGTTTCTCTCCATCTAAGGAGCTTTGGGGGAACATTTT-TAGTTCCCCTACCACCAAGCCTT 98
Rn TGAGGTTTCCT---CCGTGGTTTCTCTCCATCTAAAGGGCTTTGGGGAACATTTT-TAATCCCCCTACCACCATGCCTT 98
Mr TGAGC-CTCCT---CTGTGGTCTCTGTCCATCTATCGGGGTCTCTGGGAACATTTT-TAGTTCCCTCACCACCATGCCTT 98

```
           *  **                    *           *
    Bt  ATGGCATATTTC-TTGGAAAAAATTACACCAAAAAT--TCATAAAATATTTTT  146
    Cf  ATGCCGTATTTCTTTAAGAAAAT--TCACCCAAATT--CCATAAAATA-TCTT  143
    Dn  AGGGC--ATTTCCCTCAAAGAGGAGCCATGAAGATTA--CCTAAAATGTT-TT  143
    Ec  ATGGCATATTTCTTTAGAAAAA--TTCACCAAAATT--CCATAAAATATTTTT  145
    Hs  ATGGCGTATTTCTTTAAAA---AAATCACCTAAATT--CCATAAAATA-TTTT  144
    La  G-GGCATATTTCTCTGAAAAGCAAATCACCGAAATTAGTTCTAAAAC--TAGG  140
    Oc  ATGGGGCATTTCTTAAGAAAA---ATCACCAAAATGA--CATAAAGGGTTTTA  146
    Sa  CTGGGGTACTTATTTTTAAGGACCATC-CTAAATT---CCATAATATACTTGA  139
    Ss  ATGGCATATTTCTTTAAAAAAAAATCCACCAAAAAT--TCATAAAATGTTTTA  147
    Cp  ATAGGGTACTTAGCAAACAAAAGCAAT-CAGAATT---ACATAAAATATTT-A  145
    Mm  ATGGCTTATTTAAGAAAACA------TATCAAAATT--CCACGAGAT-----T  138
    Rn  ATGGTGTATTTAAGAAAACA------TATCAAAATT--ACATAAGAT-----T  138
    Mr  ATGGTGTAATTAAGAAAATA------TATCAAGATT--CTATAATAT-----T  138
```

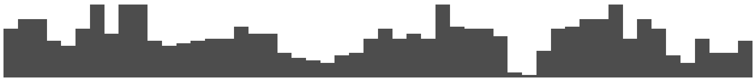

Supplement: Figure S1 — The alignment of 5′ regulatory regions and parts of the first exon of Xist in different mammalian lineages. (Bt) Bos taurus; (Cf) Canis familiaris; (Dn) Dasypus novemcinctus (armadillo); (Ec) Equus caballus (horse); (Hs) Homo sapiens; (La) Loxodonta africana (elephant); (Oc) Oryctolagus cuniculus (rabbit); (Sa) Sorex araneus (shrew); (Ss) Sus scrofa (pig), (Cp) Cavia porcellus (guinea pig), (Mm) Mus musculus; (Rn) Rattus norvegicus; (Mr) M. rossiaemeridionalis (vole). Conserved regions are shown with red frames; arrow indicates Xist transcription start site. (PDF) [file pone.0033994.s001.pdf]
