# Supplementary figures and images for: A Regulatory Potential of the Xist Gene Promoter in Vole M. rossiaemeridionalis
Source: PLoS One. 2012 May 11;7(5):e33994. doi: 10.1371/journal.pone.0033994 (PMC3350511; doi:10.1371/journal.pone.0033994)

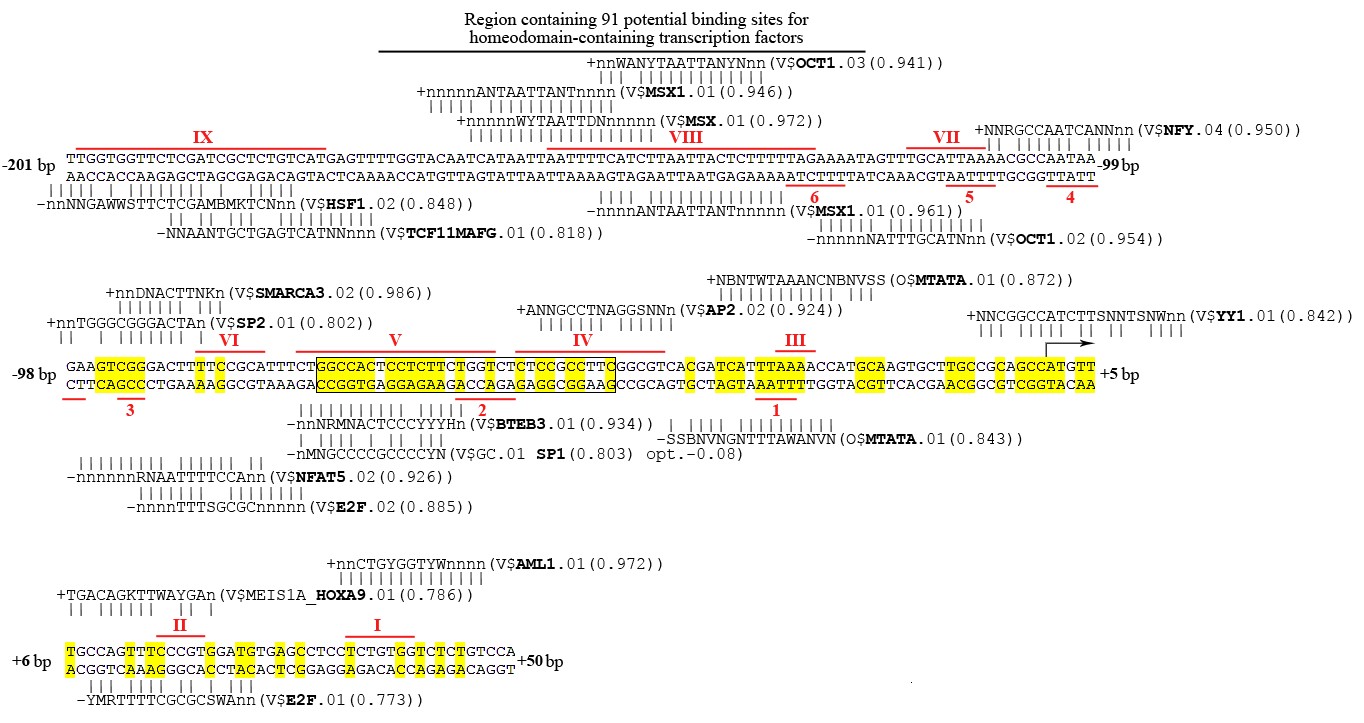

Supplement: Figure S2 — Computational analysis of the promoter region of the M. rossiaemeridionalis Xist gene. Consensuses of several identified potential transcription factor binding sites are shown above and below the nucleotide sequence. The sequence corresponding to the first conserved region, CNS1, is framed with a rectangle. Footprints are shown with red lines and numerals. Roman numerals denote the protected DNA motifs identified in the (+)-strand and Arabic numerals, in the (−)-strand; arrow shows the transcription start site. The nucleotides conserved for vole, human, cow, dog, horse, and rabbit are shown in yellow. (TIF) [file pone.0033994.s002.tif]

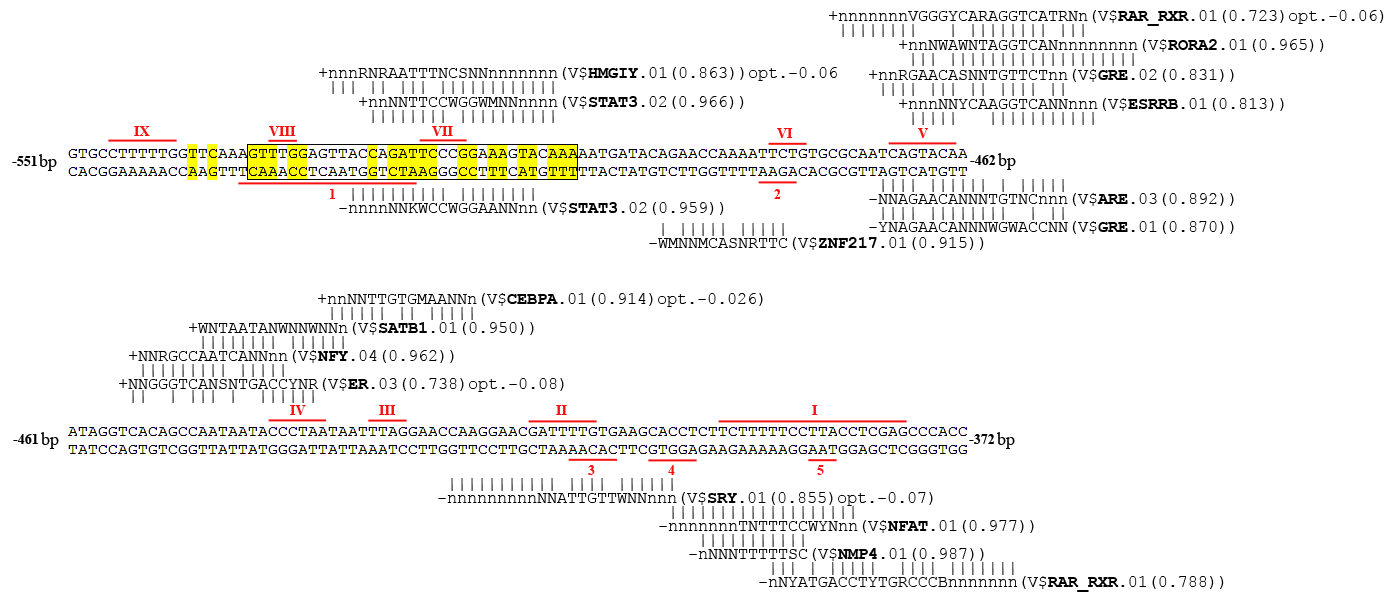

Supplement: Figure S3 — Computational analysis of the second conserved region (CNS2) and the adjacent sequences in the 5′ region of the M. rossiaemeridionalis Xist gene. Consensuses of several identified potential transcription factor binding sites are shown above and below the nucleotide sequence. The sequence corresponding to CNS2 is framed with a rectangle. Footprints are shown with red lines and numerals. Roman numerals denote the protected DNA motifs identified in the (+)-strand and Arabic numerals, in the (−)-strand. In CNS2, the nucleotides conserved for vole, human, cow, dog, horse, and rabbit are shown in yellow. (TIF) [file pone.0033994.s003.tif]

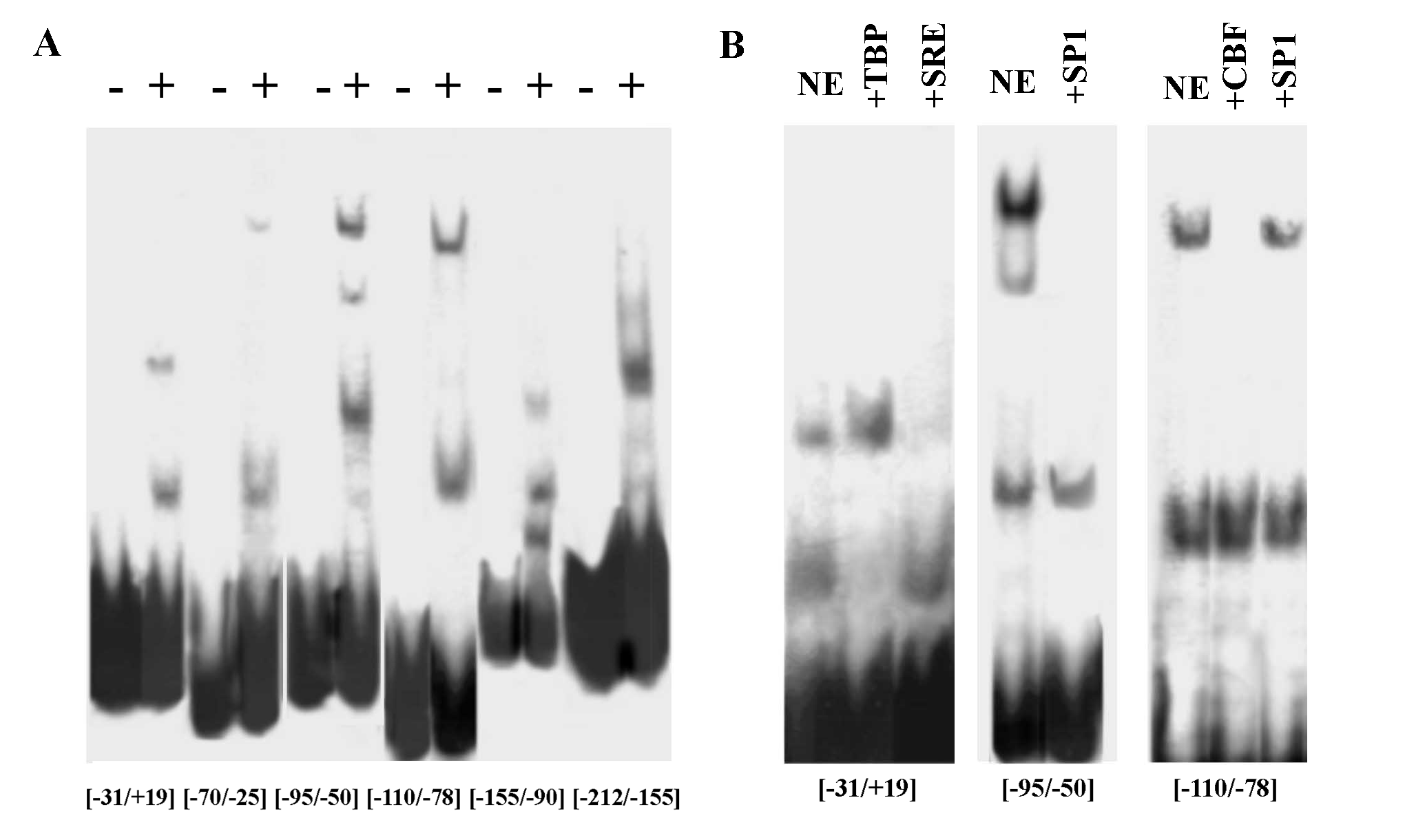

Supplement: Figure S4 — (A) Interaction of the vole Xist minimal promoter with the liver nuclear extract. (−) [γ-32P]ATP labeled DNA fragments (0.05 pmol) without incubation with the nuclear extract (NE). (+) [γ-32P]ATP labeled DNA fragments (0.05 pmol) incubated with NE (5–10 µg) (Buffer: 10 mM HEPES pH 7.6, 40 mM KCl, 2 mM MgCl2, 0.1 mM EDTA, 0.5 mM PMSF, 1 mM DTT, 10% glycerol). The regions [−31/+19 bp] and [−70/−25 bp] of the vole Xist promoter were obtained by hydrolysis of a PCR product with Sau3AI. The primers used were 5′-cacgggaaactggcaaacat-3′ and 5′-cactcctcttctggtctct-3′. The [−95/−50 bp] fragment was amplificated with 5′-gaagtcgggacttttccgc-3′ and 5′-agagaccagaagaggagtg-3′. The promoter region [−110/−78 bp] was amplificated with 5′-taaaacgccaataagaag-3′ and 5′-gcggaaaagtcccgacttc-3′ primers. The fragments [−155/−90 bp] and [−212/−155 bp] were generated by hydrolysis of a PCR product with VspI. The primers used were 5′-cccgacttcttattggcgtttta-3′ and 5′-atatacaaatttggtggttctc-3′. (B) Competitive inhibition of EMSA. (NE) [γ-32P]ATP labeled DNA fragments (0.05 pmol) with nuclear extract only. In the other reactions, unlabeled double-stranded oligonucleotides (5 pmol; 100x) containing binding sites for a number of transcription factors were added. (TBP) 5′-gcagagcatataaggtgaggtagga-3′ oligonucleotides with the TBP binding site («Promega»); (SRE) 5′-cagtacaggatgtccatattaggacacatctgcgt-3′ oligonucleotides with the YY1 binding SRE-element [23]; (SP1) 5′-attcgatcggggcggggcgagc-3′ oligonucleotides with the Sp1 binding site («Promega»); (CBF) 5′-cgtctccaccaatgggagggctggc-3′ oligonucleotides with the CBF binding site [24]. The fragment [−31/+19 bp] of the vole Xist promoter gave two retardation bands in EMSA. An addition of unlabeled the “TBP” oligonucleotides resulted in a loss of the band corresponding to a more electrophoretic mobile complex. When added the “SRE” oligonucleotides a less mobile complex disappeared. The fragment [−95/−50 bp] comprises a potential Sp1 binding site. In EMS [file pone.0033994.s004.tif]

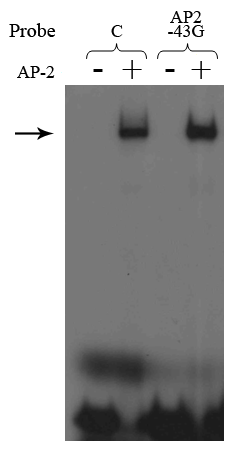

Supplement: Figure S5 — AP2 binding to the vole Xist promoter in vitro. C, DNA probe containing consensus binding site for AP2 (positive control); AP2–43G, DNA probe containing the [−54/−36 bp] region with guanine at position −43 bp; (+) denotes the lanes with the DNA probes incubated with AP2 extract and (−) without AP2 extract. Arrow indicates the specific complex formed by AP2 binding to DNA probe. (TIF) [file pone.0033994.s005.tif]

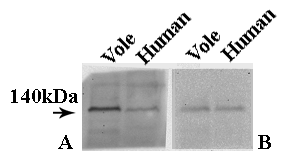

Supplement: Figure S6 — Western blot analysis of extracts from vole and human fibroblasts using CTCF antibody - Cell Signaling #2899 (A), Upstate #07-729 (B). (TIF) [file pone.0033994.s006.tif]

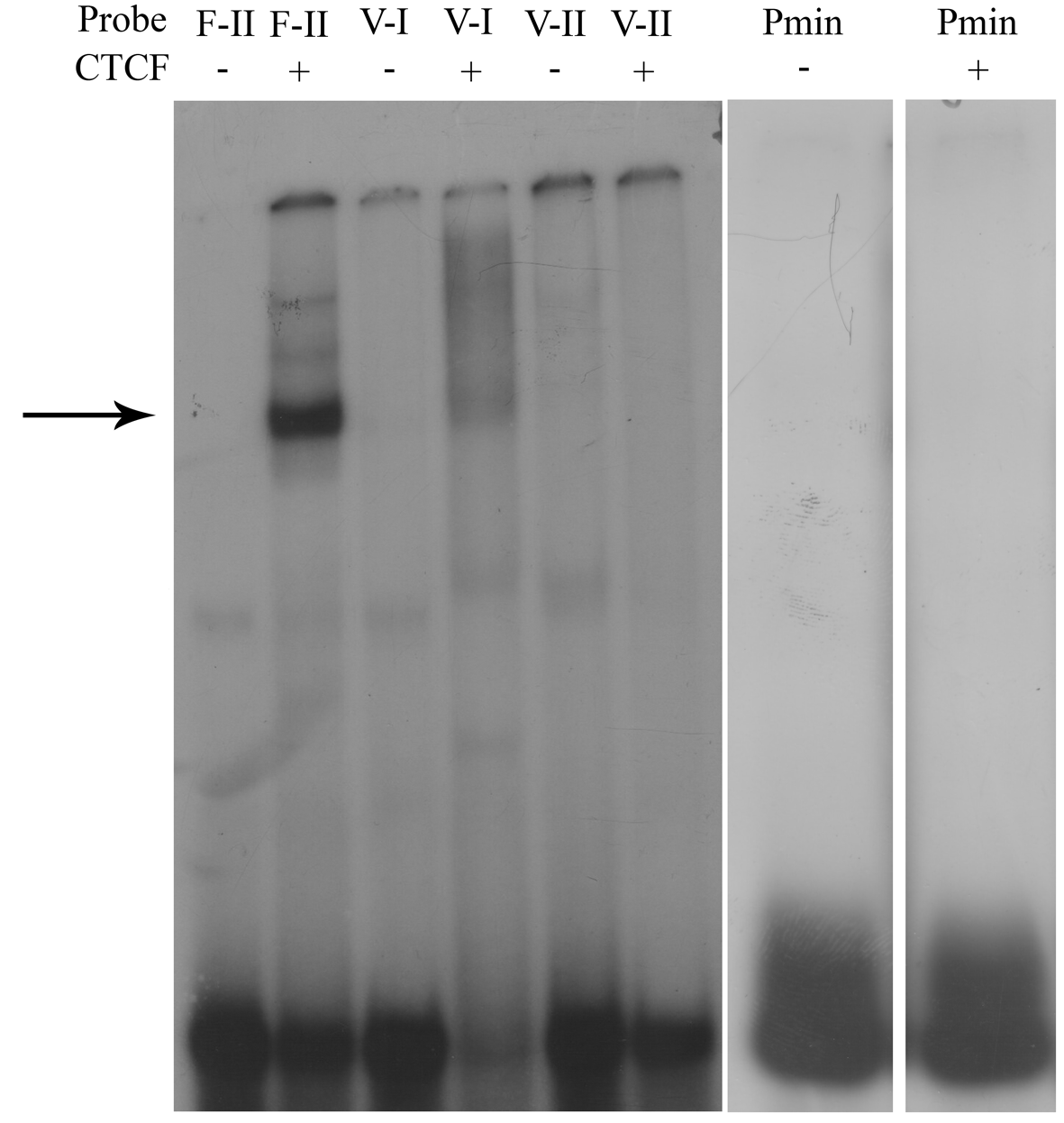

Supplement: Figure S7 — EMSA analysis of the CTCF transcription factor binding to the Xist minimal promoter. (+) indicates the lanes with DNA probes incubated with CTCF and (−) without CTCF. Arrow denotes the specific complex formed by CTCF binding to F-II probe. (TIF) [file pone.0033994.s007.tif]

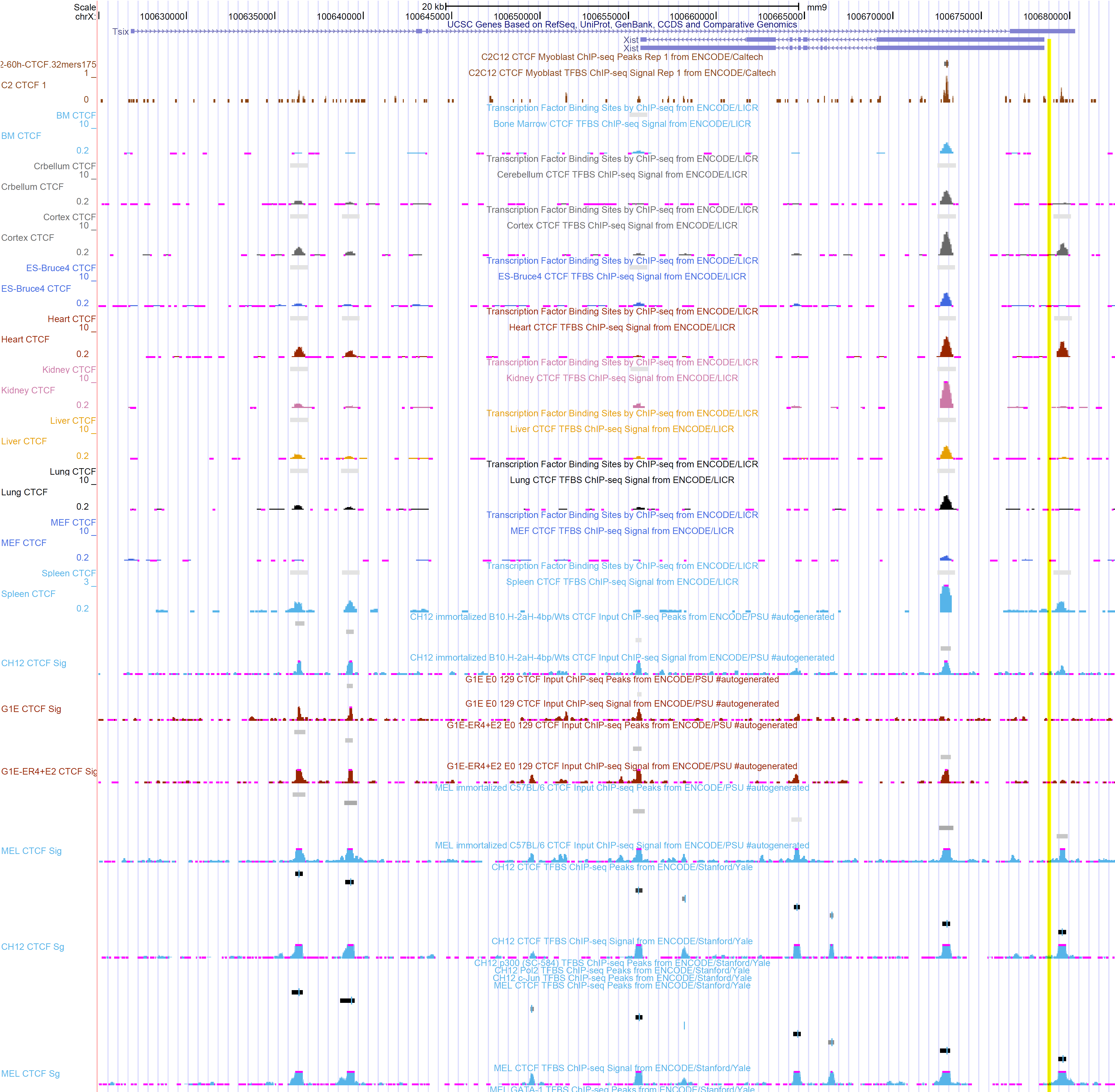

Supplement: Figure S8 — Chip-seq data on CTCF binding in mouse (UCSC genome browser). Significant sites are shown with rectangles. The Xist minimal promoter is indicated in yellow. (TIFF) [file pone.0033994.s008.tif]

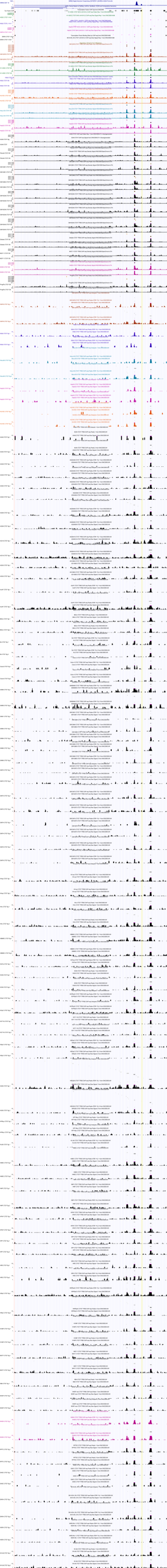

Supplement: Figure S9 — Chip-seq data on CTCF binding in human (UCSC genome browser). Significant sites are shown with rectangles. The Xist minimal promoter is indicated in yellow. (PDF) [file pone.0033994.s009.pdf]
