# Supplementary material for: A Regulatory Potential of the Xist Gene Promoter in Vole M. rossiaemeridionalis
Source: PLoS One. 2012 May 11;7(5):e33994. doi: 10.1371/journal.pone.0033994 (PMC3350511; doi:10.1371/journal.pone.0033994)
Supplement: Table S1 — Description of the transcription factors with potential binding sites identified in the 5′ region of the M. rossiaemeridionalis Xist. (DOC) [file pone.0033994.s010.doc]

**Table S1**. Description of the identified potential transcription factors from the 5’ region of the *M. rossiaemeridionalis Xist* gene

| Reporter constructs | Luciferase activity | Footprint protected region | Identified factor | Description |
| --- | --- | --- | --- | --- |
| **CNS1** |  |  |  |  |
| pCx1  [-1/+67] | 0.02 |  | YY1 | YY1 is belonging to the GLI-Kruppel class of zinc finger proteins. The protein is involved in repressing and activating a diverse number of promoters. YY1 may direct histone deacetylases and histone acetyltransferases to a promoter in order to activate or repress the promoter, thus implicating histone modification in the function of YY1 |
| pCx2  [-50/+67] | 0.02 | III/1 | TBP | General transcription factor that functions at the core of the DNA-binding multiprotein factor TFIID |
| pCx3  [-100/+67] | 60.2 | IV;V | Sp1, | SP1 is a transcription factor that binds to GC box promoters elements and selectively activates mRNA synthesis from genes that contain functional recognition sites. |
|  |  |  | AP2 | AP2 activate genes involved in a large spectrum of important biological functions |
|  |  |  | BTEB3/ KLF13 | BTEB3 (Basic Transcription Element Binding protein 3). KLF13 (Kruppel-like factors) contain 3 classical zinc finger DNA-binding domains These transcription factors bind to GC-rich sequences and related GT and CACCC boxes |
| pCx4  [-150/+67] | 39.7 | 4 | NFY | Repress transcription either acting directly or via chromatin modification in the *Xist* promoter region on the active X chromosome [1] |
|  |  | VII/5 | Oct1 | recruit corepressors [2]. |
|  |  | VIII/6 | Msx1 | Msx1 (Msh homeobox 1)can interact with other transcription factors (SP1, homeodomain-containing factors) |
| pCx5  [-200/+67] | 34.8 | IX | TCF11/  MAFG | Mafs are basic leucine zipper (bZIP)proteins behave as transcriptional repressors when they dimerize among themselves. They serve as transcriptional activators by dimerizing with other (usually larger) bZip proteins and recruiting them to specific DNA-binding sites. |
| pCx6  [-250/+67] | 65.2 |  | Jarid2 | ARID (AT rich interactive domen factor)/MRF2(modulator recognition factor) family; Regulator of histone methyltransferase complexes that plays an essential role in embryonic development. |
| pCx7  [-300/+67] | 24.4 |  |  |  |
| pCx8  [-350/+67] | 20.5 |  | Oct1 | recruit corepressors [2]. |
| **CNS2** |  |  |  |  |
| pCx9  [-400/+67] | 51.0 | I/5 | NFAT, | NMP4(Nuclear Matrix Protein 4),: transcription activator and regulates gene expression in various cell types [3]. |
|  |  |  | NMP4 | NFAT (Nuclear Factor of Activated  T-cells): activator of gene expression; tissue-specific. Therefore, the potential NFAT transcription factor binding site in the 5’ region of vole *Xist* is most likely nonfunctional. |
|  |  |  | RAR_RXR  (Retinoic Acid  Receptor/Retinoid  X Receptor) | The heterodimer RAR_RXR in the absence of its ligand forms the complex with a corepressor and binds to the target sequence, a hormone-sensitive element. Interaction with the ligand causes dissociation of the corepressor, and RAR_RXR interacts with a coactivator, able to recruit other transcription factors and RNA polymerase II, which activates transcription of the target gene [4]. |
| pCx10  [-450/+67] | 49.0 | II/3, 4 | SRY | This transcription factor is responsible for determination of the male sex |
|  |  | III | CEBPA | (CCAAT /Enhancer-Binding Protein Alpha) activator |
|  |  | IV | NFY | Repress transcription either acting directly or via chromatin modification in the *Xist* promoter region on the active X chromosome [1] |
|  |  | IV | SATB1 | SATB1 (Special AT-rich sequence Binding protein 1) belongs to architectural proteins, which are involved in formation, maintenance, and regulation of the function of the chromatin loop domains by attaching their bases to the nuclear matrix or scaffold. |
| pCx11  [-500/+67] | 15.3 | IV | ERα | ERα (Estrogen Receptor alpha), ESRRB(EStrogen-Related Receptor Beta), AR (Androgen Receptor), GRE(Glucocorticoid Receptor).Orphan Receptor . These receptors are localized to the cytoplasm and migrate to the nucleus when interacting with the corresponding hormone via an active transport; in the nucleus, they activate or repress gene expression. |
|  |  | V | ESRRB |  |
|  |  |  | AR |  |
|  |  |  | GRE(Glucocorticoid Receptor), |  |
|  |  |  | RORA2 (Rar-related Orphan Receptor alpha | The RORα ligand is the epiphysis hormone melatonin, and this receptor is involved in regulation of immune processes, differentiation of the central nervous system, and modulation of lipid metabolism [5].. |
|  |  |  | RAR_RXR |  |
|  |  | VI/2 | Znf217 (Zinc finger protein 217), | Znf217: interacts with the corepressor CtBP (C-terminal Binding Protein) and inhibits expression of the target genes [6]. |
| pCx12  [-550/+67] | 37.8 | VII/1 | HMGA1 (High Mobility Group) | The factor HMGA1 carries an AT-hook DNA binding domain, which is involved in the interaction with AT-rich DNA, and plays the role of architectural transcription factor, which regulates expression of various genes by changing the chromatin structure and recruiting other transcription factors via protein–protein interactions. *HMGA1* is also expressed in embryonic fibroblasts; however, its expression in adult organism is found only in several cell types, such as bone marrow cells, macrophages, and tumor cells [7]. |
|  |  |  | STAT3, | STAT protein family. Involved in the signal transduction from the cell membrane to nucleus [8]. |
| pCx13  [-843/+67] | 41.3 |  |  |  |
| pCx14  [-1453/+67] | 33.5 |  | CTCF | CTCF zinc finger transcription factor. A sequence specific DNA binding protein that functions as an insulator, blocking enhancer activity. It has also been suggested to block the spreading of chromatin structure in certain instances. |

1. Peng Y, Jahroudi N (2003) The NFY transcription factor inhibits von Willebrand factor promoter activation in non-endothelial cells through recruitment of histone deacetylases. J Biol Chem 278: 8385-8394.

2. Malin S, Linderson Y, Almqvist J, Ernberg I, Tallone T, et al. (2005) DNA-dependent conversion of Oct-1 and Oct-2 into transcriptional repressors by Groucho/TLE. Nucleic Acids Res 33: 4618-4625.

3. Alvarez M, Shah R, Rhodes SJ, Bidwell JP (2005) Two promoters control the mouse Nmp4/CIZ transcription factor gene. Gene 347: 43-54.

4. Allenby G, Bocquel MT, Saunders M, Kazmer S, Speck J, et al. (1993) Retinoic acid receptors and retinoid X receptors: interactions with endogenous retinoic acids. Proc Natl Acad Sci U S A 90: 30-34.

5. Jetten AM (2004) Recent advances in the mechanisms of action and physiological functions of the retinoid-related orphan receptors (RORs). Curr Drug Targets Inflamm Allergy 3: 395-412.

6. Quinlan KG, Nardini M, Verger A, Francescato P, Yaswen P, et al. (2006) Specific recognition of ZNF217 and other zinc finger proteins at a surface groove of C-terminal binding proteins. Mol Cell Biol 26: 8159-8172.

7. Cleynen I, Van de Ven WJ (2008) The HMGA proteins: a myriad of functions (Review). Int J Oncol 32: 289-305.

8. Calo V, Migliavacca M, Bazan V, Macaluso M, Buscemi M, et al. (2003) STAT proteins: from normal control of cellular events to tumorigenesis. J Cell Physiol 197: 157-168.
