# Supplementary material for: A Regulatory Potential of the Xist Gene Promoter in Vole M. rossiaemeridionalis
Source: PLoS One. 2012 May 11;7(5):e33994. doi: 10.1371/journal.pone.0033994 (PMC3350511; doi:10.1371/journal.pone.0033994)
Supplement: Table S2 — Nucleotide sequences used for obtaining DNA probes for EMSA. (DOC) [file pone.0033994.s011.doc]

Table S2. Nucleotide sequences used for obtaining DNA probes for EMSA

| Probe | Oligonucleotide sequences, 5’→3’ |
| --- | --- |
| F-II [1] | Up: CCCCC-AGGGA-TGTAA-TTACG-TCCCT-CCCCC-GCTAG-GGGGC-AGCAG  Down: GAATC-TGCTG-CCCCC-TAGCG-GGGGA-GGGAC-GTAAT-TACAT-CCCTG-GGGG |
| V-I | Up: TCTGG-TCTCT-CCGCC-TTCGG-CGTCA-CGATC-ATTTA-AAACC-ATGCA  Down: GAATA-GTGGG-GTTTT-AAATG-ATCGT-GACGC-CGAAG-GCGGA-GAGAC-CAGA |
| V-II | Up: GCCTT-CGGCG-TCACG-ATCAT-TTAAA-ACCAT-GCAAG-TGCTT-GCCGC  Down: GAATG-ACCAG-AAGAG-GAGTG-GGGTT-TTAAA-TGATC-GTGAC-GCCGA-AGGC |
| AP2K | Up: TCACG-GCCCC-AGGCG-TCAC  Down: GAATG-TGACG-CCTGG-GGCCG-TGA |
| AP2-43G | Up: TCTCC-GCCTT-CGGCG-TCAC  Down: GAATG-TGACG-CCGAA-GGCGG-AGA |

1. Renda M, Baglivo I, Burgess-Beusse B, Esposito S, Fattorusso R, et al. (2007) Critical DNA binding interactions of the insulator protein CTCF: a small number of zinc fingers mediate strong binding, and a single finger-DNA interaction controls binding at imprinted loci. J Biol Chem 282: 33336-33345.
